# Repeated genetic adaptation to altitude in two tropical butterflies

**DOI:** 10.1038/s41467-022-32316-x

**Published:** 2022-08-09

**Authors:** Gabriela Montejo-Kovacevich, Joana I. Meier, Caroline N. Bacquet, Ian A. Warren, Yingguang Frank Chan, Marek Kucka, Camilo Salazar, Nicol Rueda-M, Stephen H. Montgomery, W. Owen McMillan, Krzysztof M. Kozak, Nicola J. Nadeau, Simon H. Martin, Chris D. Jiggins

**Affiliations:** 1 Department of Zoology, University of Cambridge, Cambridge, CB2 3EJ UK; 2 St John’s College, University of Cambridge, Cambridge, CB2 3EJ UK; 3 Universidad Regional Amazónica Ikiam, Tena, Ecuador; 4 Friedrich Miescher Laboratory of the Max Planck Society, Max Planck Ring 9, 72076 Tübingen, Germany; 5 Department of Biology, Faculty of Natural Sciences, Universidad del Rosario, Carrera 24 # 63C-69, Bogotá, 111221 Colombia; 6 School of Biological Sciences, University of Bristol, Bristol, UK; 7 Smithsonian Tropical Research Institute, Panamá, Panamá; 8 Ecology and Evolutionary Biology, School of Biosciences, University of Sheffield, Sheffield, S10 2TN UK; 9 Institute of Evolutionary Biology, University of Edinburgh, Edinburgh, UK

**Keywords:** Population genetics, Evolutionary genetics, Genetic variation, Evolutionary biology

## Abstract

Repeated evolution can provide insight into the mechanisms that facilitate adaptation to novel or changing environments. Here we study adaptation to altitude in two tropical butterflies, *Heliconius erato* and *H. melpomene*, which have repeatedly and independently adapted to montane habitats on either side of the Andes. We sequenced 518 whole genomes from altitudinal transects and found many regions differentiated between highland (~ 1200 m) and lowland (~ 200 m) populations. We show repeated genetic differentiation across replicate populations within species, including allopatric comparisons. In contrast, there is little molecular parallelism between the two species. By sampling five close relatives, we find that a large proportion of divergent regions identified within species have arisen from standing variation and putative adaptive introgression from high-altitude specialist species. Taken together our study supports a role for both standing genetic variation and gene flow from independently adapted species in promoting parallel local adaptation to the environment.

## Abstract (web summary)

Here, the authors study adaptation to altitude in 518 whole genomes from two species of tropical butterflies. They find repeated genetic differentiation within species, little molecular parallelism between these species, and introgression from closely related species, concluding that standing genetic variation promotes parallel local adaptation.

## Introduction

Understanding how organisms adapt to the environment is a central goal of evolutionary biology and highly relevant given the pace of global change. One approach is to explore the repeatability of local adaptation in the wild in order to understand whether phenotypic and genetic changes are predictable. On the one hand, repeated adaptation to similar environments can act as a ‘natural experiment’ and provide the means to identify the targets of selection, by distinguishing locally adaptive from neutral or globally beneficial changes^1^. On the other hand, these scenarios can allow us to test whether the same loci are repeatedly targeted across populations and species^2^. Despite many studies reporting repeated adaptation involving the same genes or alleles across lineages^3–5^, which we here term ‘molecular parallelism’, we know relatively little about the evolutionary mechanisms that facilitate this process.

**Fig. 1:**
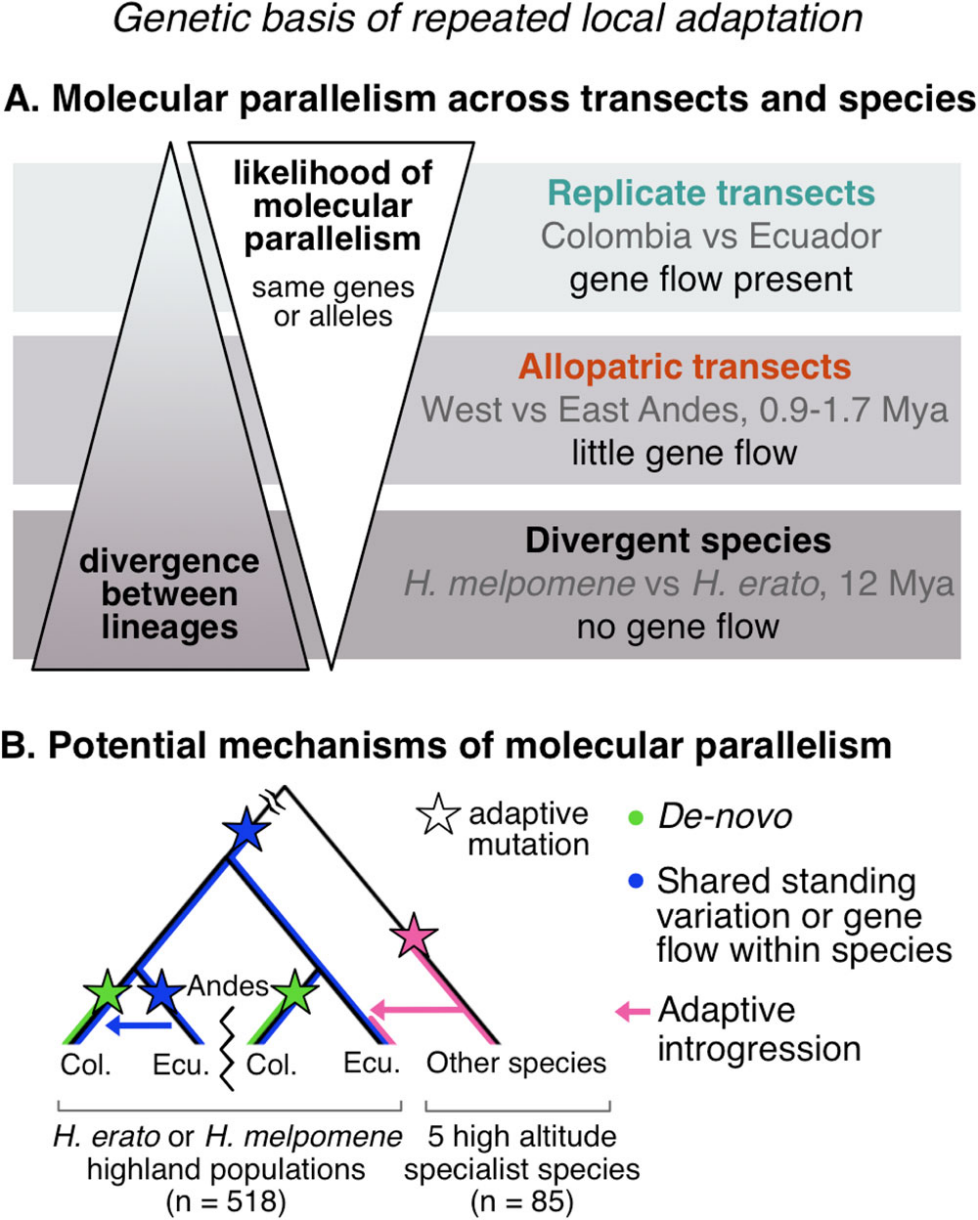
The study of repeated adaptation to the environment and the mechanisms potentially facilitating it. **A** We hypothesise that increasing divergence between the lineages under study reduces the likelihood of molecular parallelism (same genes or alleles) underlying repeated adaptation to the environment. In this study, we test this hypothesis by sampling replicate (within sides of the Andes) or allopatric (across sides of the Andes) altitudinal transects of the same species, i.e., connected via gene flow or not (divergence times indicated in Million years ago, Mya), and all replicated in two divergent *Heliconius* species. **B** Three main mechanisms can give rise to the genetic variation upon which selection acts repeatedly, giving rise to molecular parallelism: (i) adaptive *de-novo* mutations independently arise in two or more lineages, (ii) existing shared standing variation is repeatedly selected across lineages or shared via gene flow within species, and (iii) adaptive alleles are shared via gene flow across species (adaptive introgression). We tested for the relative importance of these mechanisms with a range of analyses on the relevant transect comparisons. Illustrative tree including four highland populations from four transects (with the Andes preventing gene flow between replicate transects across sides) of our focal species, either *H. erato* or *H. melpomene*, and a lineage of a related high-altitude specialist species from which adaptive introgression is plausible. Sample sizes for the full datasets (including lowland populations when present) are shown in brackets.

Three main mechanisms can give rise to molecular parallelism in repeated adaptation (Fig. 1). Genetic variation upon which selection repeatedly acts may arise via independent mutations at the same gene or locus^6^. Beneficial variants may be recruited from ancestral standing variation^7^ or shared across populations of the same species via migration and gene flow^8^. Lastly, gene flow between species can facilitate the introgression of adaptive alleles^9–11^. A combination of these mechanisms may also be at play, for instance the high altitude adaptation Tibetan-EPAS1 haplotype was introgressed from Denisovan hominins but remained as neutral standing variation before positive selection occurred^12^.

The likelihood of molecular parallelism and the relative importance of each mechanism may be largely dependent on the divergence between the lineages under study (Fig. 1), but this has seldom been empirically tested^3^. For instance, populations that diverged recently and retain a large pool of standing genetic variation tend to reuse pre-existing alleles during repeated adaptation, as seen in freshwater adaptation in sticklebacks^13^, crypsis in beach mice^14^, or coastal ecotypes of bottlenose dolphins^7^. Similarly, organisms that readily hybridise in the wild are more likely to share beneficial alleles via adaptive introgression. This allows populations to rapidly adapt to, for instance, novel anthropogenic stressors such as pollutants^15^ or insecticides^16^.

Anthropogenic change is forcing organisms to move, adapt, or die, with many predicted to expand their ranges towards the highlands to escape warming and degrading lowland habitats^17^. We know very little about the genomics or predictability of adaptation to altitude, especially in the tropical insects despite making up about half of all described species (but see)^18–20^. The type and genomic architecture of the trait under study may also determine its predictability^21^. Phenotypes controlled by few, large-effect loci typically show predictable genetic paths of evolution, such as melanic colouration in mammals, fish, and birds^22–25^, perhaps due to selective constraints on genetic pathways^21,26,27^. Organisms adapting to complex environmental challenges that face multifarious selective regimes may show less predictable patterns, with functional redundancy among genes allowing for different combinations of alleles to achieve similar phenotypes^28,29^. Thus, understanding the relative importance of these mechanisms in determining the predictability of adaptation to the environment could inform future research into conservation strategies to protect biodiversity^30^.

Here, we study the genetic basis of repeated adaptation to altitude in two divergent *Heliconius* tropical butterflies, *H. erato* and *H. melpomene*. These aposematic, toxic species have very wide ranges and co-mimic each other across South America, commonly found from sea level to around 1600 m on both sides of the Andes^31^. In contrast, most other species in this genus have specialised to either highland Andean or lowland Amazonian habitats, with topography and climate shown to correlate with speciation rates across the clade^32^. The biogeography of these species’ ancestral ranges across the Andes is uncertain, with multiple plausible adaptive histories: ancestral adaptation to the highlands on one side of the Andes followed by expansion, gene flow of adaptive alleles between sides of the Andes, or fully independent in situ adaptation to altitude on either side of the Andes.

A recent study in this system showed that even a modest 800 m change in elevation in the Ecuadorian Andes leads to strong environmental differences, with temperatures in the lowlands more variable and, on average, 4 °C hotter, as well as threefold increase in the ‘drying power of the air’ (Vapour Pressure Deficit) which correlates with desiccation risk in ectotherms^33^. Phenotypic differences between highland and lowland populations of *Heliconius* have recently been identified, such as in wing shape^34,35^ and heat tolerance^33^, and are of similar magnitude in *H. erato* and *H. melpomene*. Thus, the observed environmental and phenotypic differences point towards an important role of local adaptation enabling these two species to inhabit wide altitudinal ranges.

We search for signatures of local adaptation to montane habitats with extensive sampling that harnesses the power of natural spatial replication within and across sides of the Andes, to assess the extent of molecular parallelism in adaptation to montane habitats at the upper end of the species altitudinal range (∼1200 m). We quantify parallelism at multiple levels of divergence: (i) replicate three-population transects within sides of the Andes connected via gene flow, (ii) trans-Andean allopatric three-population transects with no gene flow at these latitudes (but potential for gene flow in other regions), and (iii) two species that diverged 12 million years ago (Fig. 1A). Furthermore, we test whether the same haplotypes are under selection across transects and search for the mutational origin of candidate adaptive alleles (Fig. 1B). Overall, this large empirical study deepens our understanding of how organisms adapt to the environment and identifies both standing genetic variation and adaptive introgression from pre-adapted species as important mechanisms facilitating local adaptation.

## Results and discussion

### Divergence and diversity across elevations and transects

**Fig. 2:**
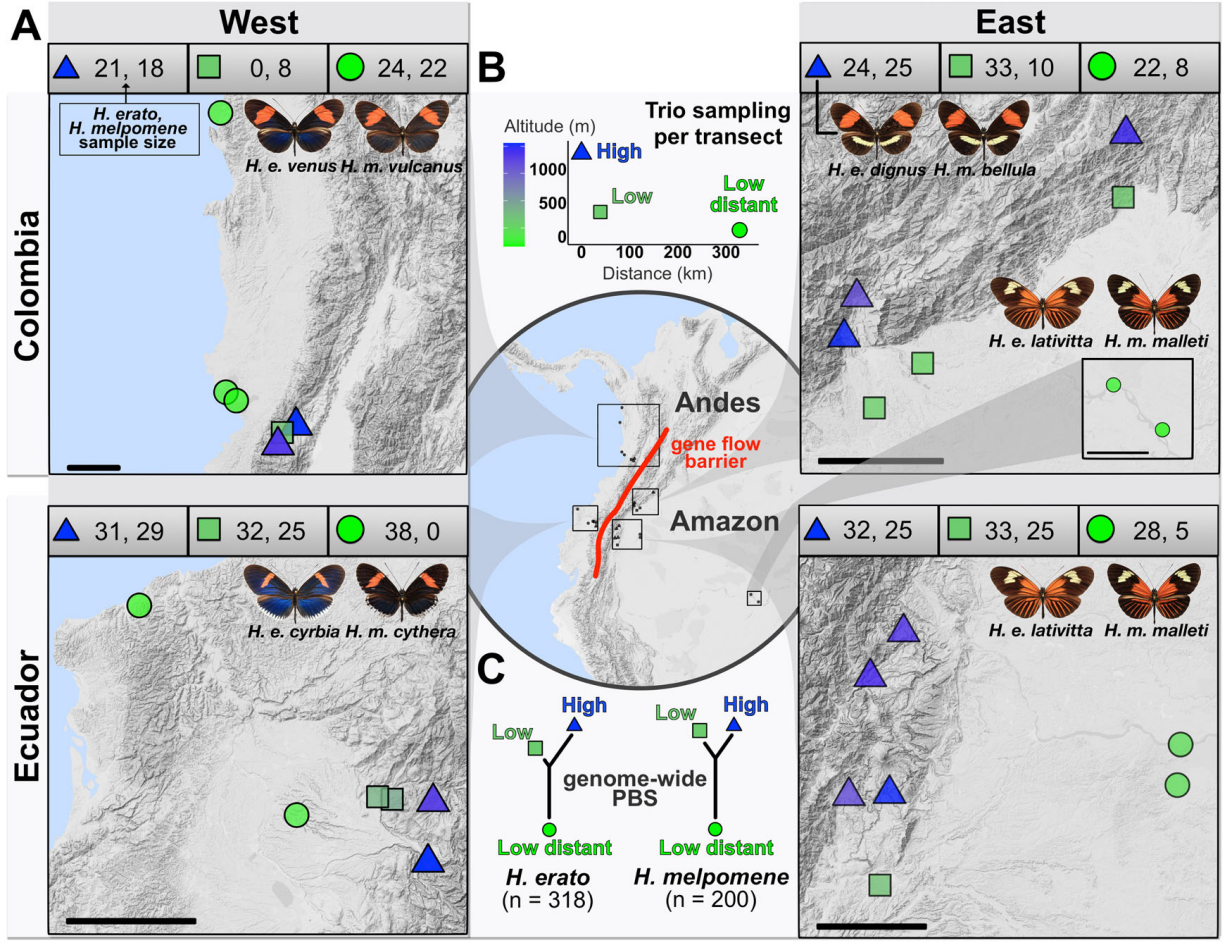
Sampling design. **A** Elevation map of the 30 populations sampled for this study in four geographical transects (Colombia West/East, Ecuador West/East). More details of each population can be found in Supplementary Table 1, number of whole-genome sequences included per altitude and transect is indicated above each map (*H. erato*, *H. melpomene*). Source data are provided as a Source Data file and Supplementary Data 1. The black scale bar represents 25 km. Maps and *Heliconius* subspecies present are depicted per transect (photo credit: C.D.J. McGuire Center for Lepidoptera and Biodiversity, Florida Museum of Natural History; Map data ©2019 Google obtained via RgoogleMaps package^120^), note that only Colombia East has different subspecies in the highlands compared to the lowlands. **B** Plot depicts trio sampling scheme, with mean altitudinal and geographical distance of the three population types (high, low, low distant) for both species. **C** Mean genome-wide Population Branch Statistic (PBS) trees averaged across the four transects per species and respective total sample size in brackets. Source data are provided in the Source Data file.

To study adaptation to altitude in *H. erato* and *H. melpomene*, we used whole-genome data from 518 re-sequenced individuals, 444 of which were sequenced for this study. Samples were collected from 111 different locations, which we grouped into 30 populations, corresponding to four three-population transects: Colombia West/East, and Ecuador West/East (Fig. 2, Supplementary Table 1). In each three-population transect, populations were either in the highlands (~1200 m) or lowlands (~200 m), itself divided into nearby or distant lowland sites (Fig. 2A). The Andes act as a barrier to gene flow at these latitudes, with populations on opposite sides of the Andes thought to have split ~0.9 and ~1.7 million years ago for *H. erato* and *H. melpomene*, respectively^36–38^ (for natural history see Supplementary Note 1). Individuals of each species clustered strongly into Western and Eastern groups in genome wide PCAs (S.I., Supplementary Fig. 1). In PCAs that only included populations from replicate transects of the same side of the Andes (two per species), structuring by altitude was absent in all but one comparison, *H. erato* East, where the *H. erato* highland population in Colombia corresponds to a different colour pattern subspecies and diverged moderately from other populations (S.I., Supplementary Fig. 1). Intraspecific pairwise differentiation between populations on the same side of the Andes increased with geographic distance but was generally low (F_st_ < 0.1; S.I., Supplementary Fig. 2). The effective replication over space and extensive gene flow within transects provide a powerful setting to study the genomics of parallel adaptation to altitude in the wild.

Genetic distance between populations can be increased by environmental differences that affect dispersal or survival of locally adapted migrants^39,40^. We tested for such isolation by environment, in our case altitude, with pairwise F_st_ across all populations of the same side of the Andes and species. At similar geographic distances, genetic differentiation was higher when comparing highland vs. lowland populations than when comparing lowland vs lowland populations. Isolation by altitude could be driven by local adaptation reducing gene flow between elevations or due to many adaptive sites diverging across the genome. This difference was stronger in *H. erato* than in *H. melpomene* (S.I., Supplementary Fig. 2). F_st_ was generally highest when comparing two highland sampling sites at relatively short distances, despite using a topographically informed ‘least-cost path’ as our measure of geographic distance (Supplementary Fig. 3). This could indicate topographical barriers decreasing gene flow, beyond what was captured by the least-cost path, or local adaptation in the highlands leading to increased selection against migrants. A pattern of isolation by environment could also arise due to, for instance, different demographic histories and higher levels of inbreeding in range-edge populations^41–43^. We found no consistent differences in nucleotide diversity (π) between elevations (S.I., Supplementary Fig. 4). Tajima’s D was negative across populations, characteristic of population expansion, but generally less negative in highland populations, suggesting less pronounced expansion or more recent/ongoing contractions in the highlands (S.I., Supplementary Fig. 4). Thus, both heterogenous demographic histories and selection against locally adapted migrants across elevations may lead to genome-wide isolation by environment.

### Parallel high-altitude differentiation detected with population branch statistics

To identify genomic regions with high-altitude specific differentiation we calculated Population Branch Statistics (PBS) for three transects in each species, and F_st_ for the remaining two transects with more limited sampling (*H. erato* Colombia West, *H. melpomene* Ecuador West; Fig. 2A). PBS was originally developed to study high-altitude adaptation in humans^44^ and can distinguish between global and lineage specific differentiation by constructing a trifurcating population tree based on F_st_ that includes a geographically distant population^15,42,45–49^. By attributing fractions of differentiation to each branch, PBS identifies genomic regions disproportionally diverged in the focal population, consistent with loci positively selected in the highlands and either selected against or neutral in the lowlands^44,50^.

**Fig. 3:**
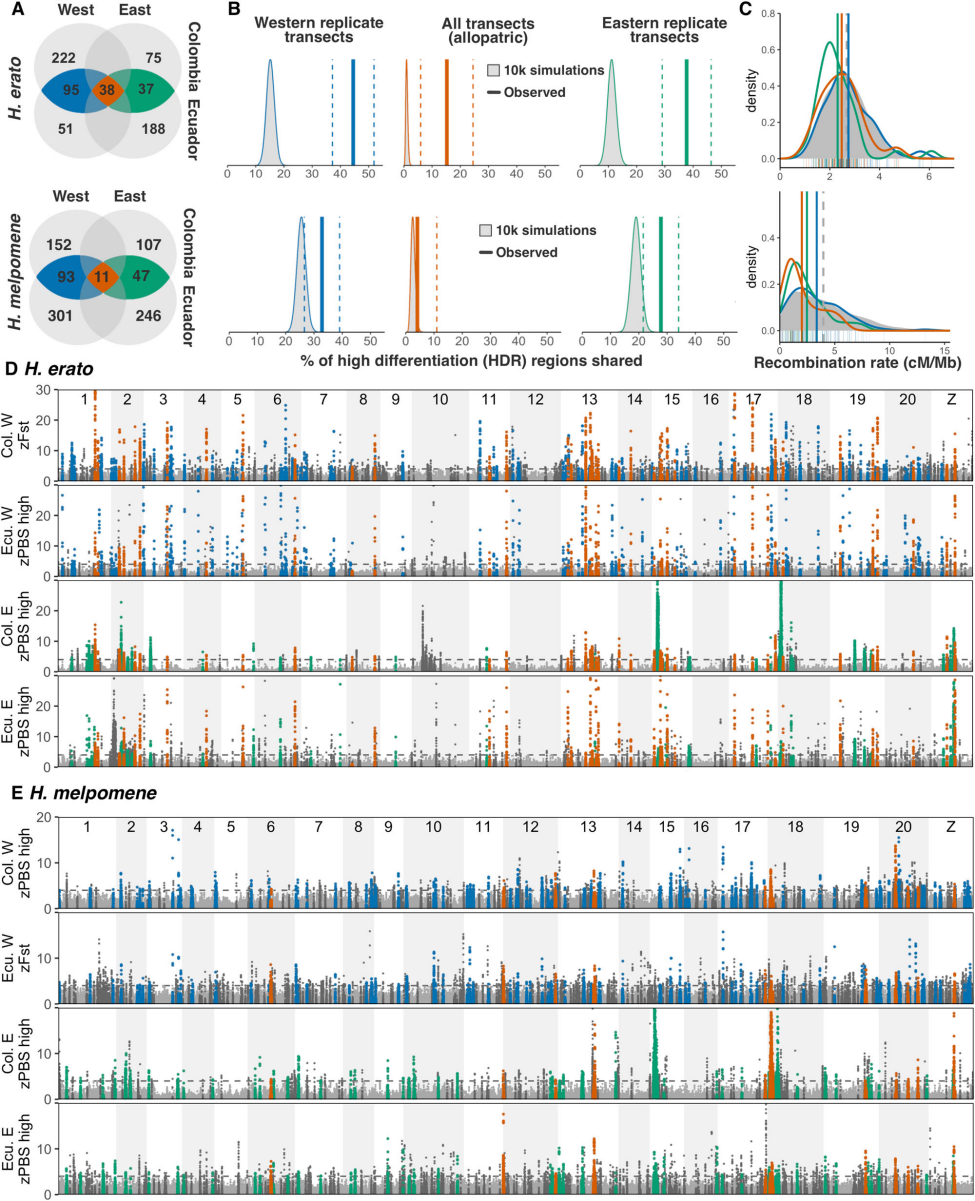
Molecular parallelism in PBS/F_st_ regions of differentiation across eight altitude transects of *H. erato* and *H. melpomene.* **A** Number of high-differentiation regions per species (HDRs, including ± 50kb buffers), in blue/green if shared across replicate transects within sides of the Andes (SHDR: blue=within West, green=within East) and in red those additionally shared across allopatric transects, i.e. shared across all four transects (also SHDR). Source data are provided in the Source Data file. **B** Vertical lines represent percentage of outlier windows shared across transects (jackknife resampling confidence intervals as dashed lines), compared to 10,000 simulations (grey distributions). **C** Density plots of local recombination rate (cM/Mb) for all genomic windows (grey), or for only windows within HDRs (coloured). **D**, **E** Patterns of highland-specific differentiation (zPBS_high_) across the genome in four transects of *H. erato* (**D**) and *H. melpomene* **E**. In the two transects where only two populations were sampled zF_st_ is presented. Horizontal dashed line indicates threshold of 4 standard deviations from the mean. HDRs private to one transect are highlighted in dark grey.

When assessing genome-wide average PBS trees the longest PBS branches corresponded to the low distant populations of both species, as expected under a model of neutral isolation by distance (Fig. 2B). *H. erato* had a consistently longer high-altitude branch compared to the lowland, but geographically nearby population, which could be indicative of increased drift or extensive local adaptation to altitude throughout the genome. Indeed, we detected many regions across the genome strongly differentiated in the highland populations across transects and species (Fig. 3). We defined High Differentiation Regions (HDRs hereafter) by adding a ± 50 kb buffer around outlier windows, i.e. those with zPBS_high_ (high-altitude branch) or zF_st_ values above 4 (standardised Z-transformed, equivalent to >4 SD^51^), and merging overlapping intervals into discrete regions. The transects for which only two populations were sampled (F_st_), had a higher number of HDRs: 400 and 405 HDRs, covering 11.4% and 17.1% of the genome for *H. erato* and *H. melpomene*, respectively (compared to, on average, 229 PBS-based HDRs covering 8%; details on S.I., Supplementary Note 2). This likely reflects the property of PBS to discern between population-specific and globally differentiated alleles.

To test for molecular parallelism in local adaptation, we assessed whether the same individual HDRs were repeatedly found in the highlands across replicate (same side of the Andes) or allopatric (opposite side of the Andes) transects within each species (Fig. 1A). In *H. erato*, 45% (±3.8 SD) and 38% (±4.4 SD) of HDRs overlapped between replicate transects within the Western and the Eastern Andes, respectively (shared HDRs, SHDRs hereafter; Fig. 3D blue, green). Of those SHDRs, more than a third were also shared across allopatric transects not connected via gene flow, 15% of the total HDRs (allopatric SHDRs hereafter; Fig. 3D red). *H. melpomene* had a slightly lower percentage of HDRs shared within sides of the Andes (West 33% ± 11 SD; East 27% ± 11 SD), but very few shared across sides of the Andes (allopatric SHDRs: 4% of the total). We then tested if the observed level of sharing was higher than expected under a null distribution of genomic regions, obtained by assessing sharing in randomly placed blocks across the genome of the same size and number as observed HDRs per transect. *H. erato* HDR sharing was higher than predicted under a null distribution in all three comparisons (replicate Eastern/Western and all transects, Fig. 3B). In *H. melpomene*, HDR sharing was significant between transects where gene flow is present (replicate Eastern/Western transects), whereas the low levels of allopatric parallelism did not differ from the null distribution (Fig. 3B, red). On average, 15.6% of SHDRs detected overlapped between species, but this fell within the null distribution given the number and size of SHDRs in each species (mean overlap = 14.06% ± 1.19).

In summary, we show that levels of parallelism are highest between populations of the same species connected by gene flow or sharing considerable ancestral variation, but low between species. Molecular parallelism within species could be facilitated by the high levels of nucleotide diversity observed^52,53^, indicating a large pool of shared variants upon which selection can repeatedly act. Furthermore, recurrent gene flow between replicate populations could facilitate the recruitment of new or standing adaptive alleles, as expected from theory^54^ and seen in other systems such as maize, *Arabidopsis*, or sticklebacks^3,8,55^. The reduced parallelism observed between allopatric populations of *H. melpomene* could be due to lower standing variation or less gene flow across the Andes, compared to *H. erato*.

The lack of significant molecular parallelism in altitude candidate loci between *H. erato* and *H. melpomene*, whose clades diverged 12 million years ago^38^, is in stark contrast with colour patterns^56^, where near-perfect local Müllerian mimics have arisen repeatedly in both species through independent mutations at a handful of conserved loci^57,58^. This difference in the extent of molecular parallelism might be explained by the nature of the trait under study: adaptation to altitude is multifarious and, as shown here, involves many genes. Genetic redundancy in polygenic adaptation may lead to evolution taking different paths to reach similar solutions, as shown for quantitative iridescent colouration in these two *Heliconius* species^59^ or in recent experimental evolution of thermal adaptation in *Drosophila*^28,60^. In contrast, the major effect loci that underlie switches in discrete colour patterning may favour genetic predictability even across divergent taxa, especially when selection is strong and reaching adaptive peaks requires large phenotypic shifts.

### Highly differentiated regions show additional signatures of selection and, generally, are not associated with low recombination rates

We tested whether highly differentiated genomic regions shared across transects (SHDRs) showed additional evidence of positive selection by computing difference in nucleotide diversity (π) across elevations (Δπ = π_high_− π_low_), deviation from neutrality in site frequency distributions (Tajima’s D), and absolute divergence (Dxy) for the same 5 kb windows. Processes other than positive selection, such as background selection, can decrease within-population diversity and thus lead to increased relative differentiation (F_st_), especially in the absence of gene flow between populations^61,62^. Thus, it is important to test for enrichment of different selection statistics to strengthen our inference of locally adaptive loci. A reduced Δπ compared to the background would indicate that a selective sweep in the highlands reduced nucleotide diversity compared to the lowlands. In selective sweep regions, Tajima’s D is expected to be low, as regions with selected haplotypes that rapidly increased in frequency would have an excess of rare alleles. Finally, absolute sequence divergence (Dxy), is expected to be high in old selective sweeps or variants, and less affected by genetic variation within populations than relative measures of differentiation such as F_st_^63^.

**Fig. 4:**
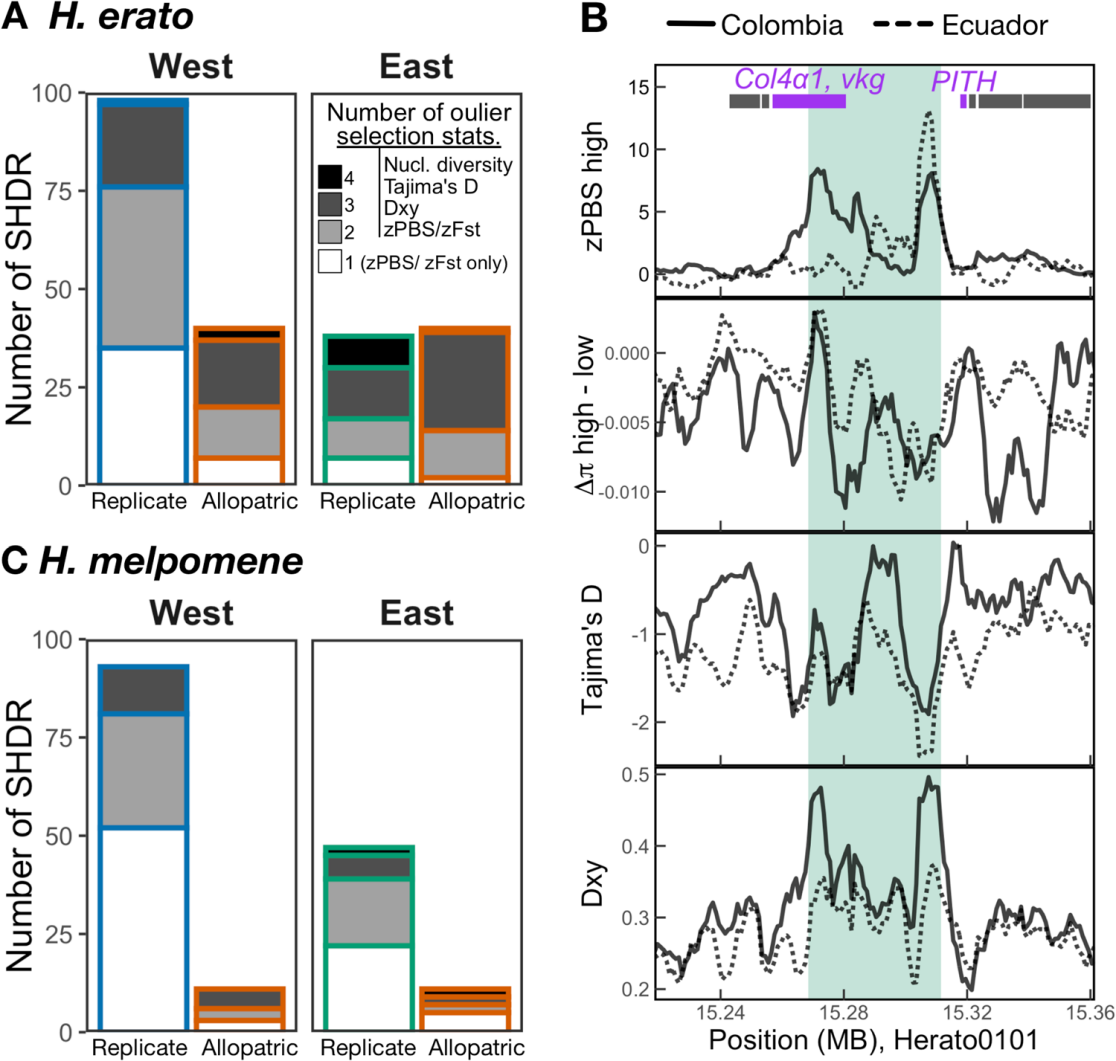
Signatures of positive selection across shared high differentiation regions (SHDRs). Number of SHDR with additional outlier selection statistics in *H. erato* (**A**) and *H. melpomene* (**C**), statistics included were nucleotide diversity difference between highlands and lowlands (Δπ), Tajima’s D, and absolute genetic differentiation (Dxy). Bars are coloured according to whether they are shared between replicate transects within sides of the Andes (blue or green) or across all transects (allopatric, in red). Shading indicates number of statistics that were above 90th percentile of simulations, white = 1 (only zPBS), light grey = 2, dark grey = 3, and black = 4 statistics. Example close-ups of regional zPBS highland values and selection statistic patterns in Eastern SHDR (**B**; number #005 in Supplementary Figs. 8–10). Each line represents the values for one of the two Eastern transects, solid line is the Colombian transect and dashed in Ecuador. In this example, all three additional selection statistics ranked as outliers among simulations. Green shading highlights the region of the eastern SHDR with zPBS_high_ > 4.

SHDRs were considered outliers for Δπ, Tajima’s D, or D_xy_, if the observed maximum or minimum values within SHDRs < 10th percentile (or > 90th in Tajima’s D) of the simulated values, obtained from 10000 permutations that randomly placed blocks of equal number and size to observed HDRs across the genome. Of the SHDRs differentiated on both Ecuadorian and Colombian transects but in one side of the Andes only, i.e. across replicate transects, on average 74% and 48% of *H. erato* and *H. melpomene* SHDRs, respectively, were outliers for at least one other statistic, in addition to zPBS/zF_st_ (Fig. 4A grey). Of the *H. erato* and *H. melpomene* SHDRs shared across all transects (allopatric SHDRs), 94% and 86% had at least one additional outlier statistic, respectively (Fig. 4A, C). In *H. erato*, SHDRs were often outliers for both, high D_xy_ and reduced Tajima’s D (36% of SHDRs with additional outlier statistics, on average). In contrast, *H. melpomene* SHDRs were rarely outliers for Tajima’s D, whereas 22-33% of them were outliers for both Δπ and D_xy_. This could point towards different selection histories in each species, with *H. erato* showing signatures of recent or ongoing selective sweeps leading to an excess of rare alleles (negative Tajima’s D). The Andean split is dated ~0.8 million years older in *H. melpomene* populations^36,37^ and their altitudinal range is wider than that of *H. erato*, which is rarely found > 1500 m at these latitudes. Thus, it is possible that *H. melpomene* SHDRs represent more ancient sweeps, reflected in the high prevalence of SHDRs outliers for D_xy_. Alternatively, high absolute sequence divergence (D_xy_) between elevations could be indicative of selected haplotypes arising through adaptive introgression from other species into the highland populations.

As an additional independent line of evidence that SHDRs are under selection, we checked for overlaps with selection statistics and altitude-associated regions obtained from an altitudinal transect in southern Ecuador on the East of the Andes, sequenced with a linked-read technology called ‘haplotagging’^64^. We found that, on average, 59% ( ± 10 SD) of Eastern SHDRs of each species overlapped with at least one additional outlier selection statistic estimated with the haplotagging dataset, whereas, as expected, fewer Western SHDRs did (32% ±8 SD, on average; S.I., Supplementary Note 3, Supplementary Fig. 5). In contrast, SHDRs shared in all transects showed high levels of overlap with haplotagging-derived selection statistics in all transects (52% ± 15 SD, on average).

We tested if SHDRs were associated with low-recombining regions. Regions of high differentiation and low recombination could be indicative of purifying selection against deleterious mutations (background selection) or maladaptive introgression^62,65,66^. Background selection has been shown to be a major driver of differentiation landscapes between populations with little gene flow^63^ but is less plausible when populations readily exchange genetic material, as in this study^67,68^. However, in low recombining regions, selection may be more efficient due to reduced effective gene flow and segregation of co-adapted alleles, and thus facilitate the maintenance of locally adaptive loci^62,69,70^. Several strongly selected *Heliconius* colour pattern loci have been previously associated with regions of low recombination (Fig. 3 chromosomes 15 and 18)^58,71^. Nevertheless, here we found that recombination rate at SHDRs did not differ from background levels, except in comparisons that included strongly selected colour pattern loci (Fig. 3C, Supplementary Fig. 6). Overall, these additional signatures of selection strongly support the action of repeated divergent selection in the highlands rather than background selection driving the differentiation detected at SHDRs.

### Known genes of interest overlap with SHDRs

We retrieved 908 and 747 genes overlapping with SHDRs in *H. erato* and *H. melpomene*, respectively. Adaptation to altitude is likely driven by a suite of selective factors such as temperature, air pressure, or habitat changes. Here, we do not distinguish among individual selective pressures, as these are often correlated and we do not have the power to tease them apart. Additionally, populations adapting to new or changing environments are unlikely to univariately respond to these pressures, with adaptive walks in the wild predicted to be as complex as the environments themselves. Nevertheless, future studies should test different selective pressures in laboratory settings and look for overlaps between genes of interest and SHDRs.

With so many potential targets of selection within SHDRs, we do not attempt to infer biological function or adaptive significance from the whole gene set. Instead, we checked for overlaps with regions recently associated with wing shape variation across an altitudinal cline of Southern Ecuador in *H. erato* and *H. melpomene*^35^. Rounder wings are generally associated with the highlands across 13 species of *Heliconius*^34^. There is also subtle but highly heritable variation in wing shape associated with altitude along the Ecuadorian Eastern transect here studied^35^. We found that five out of 12 previously identified candidate wing shape loci^35^, overlapped with SHDRs in *H. erato*, three of which corresponded to SHDRs detected in all transects. In contrast, only two wing shape loci (out of 16) overlapped with *H. melpomene* SHDRs, one of which was a SHDR shared by all transects. The number of overlaps between candidate wing shape loci and SHDRs in *H. erato* (*n* = 5) was higher than the 90th quantile of 10,000 permutations, but not in *H. melpomene* (*n* = 2; Fig. S7).

One gene on chromosome 13 stood out, *rugose*, as it was associated with wing shape in both *H. erato* and *H. melpomene*^35^ and overlapped with SHDRs shared in all transects in both species. In *Drosophila* mutants, *rugose* has been shown to affect social interactions, locomotion, and hyperactivity^72^. The highland incipient species of the *H. erato* clade, *H. himera*, has been shown to fly for more hours per day than lowland *H. erato*^73^, suggesting a potentially important role of locomotion to adapt to highland habitats. Additionally, we found that an *H. erato* Eastern SHDR (Fig. 4B) overlapped with a locus recently identified to be differentiated across many pairs of subspecies in several *Heliconius* species and shown to affect wing beat frequency in *Drosophila*^74^. Thus, future studies could focus on functionally testing some of these candidates and ascertain the potentially adaptive functions of candidate regions.

### Same haplotypes underlie parallel adaptation to altitude

**Fig. 5:**
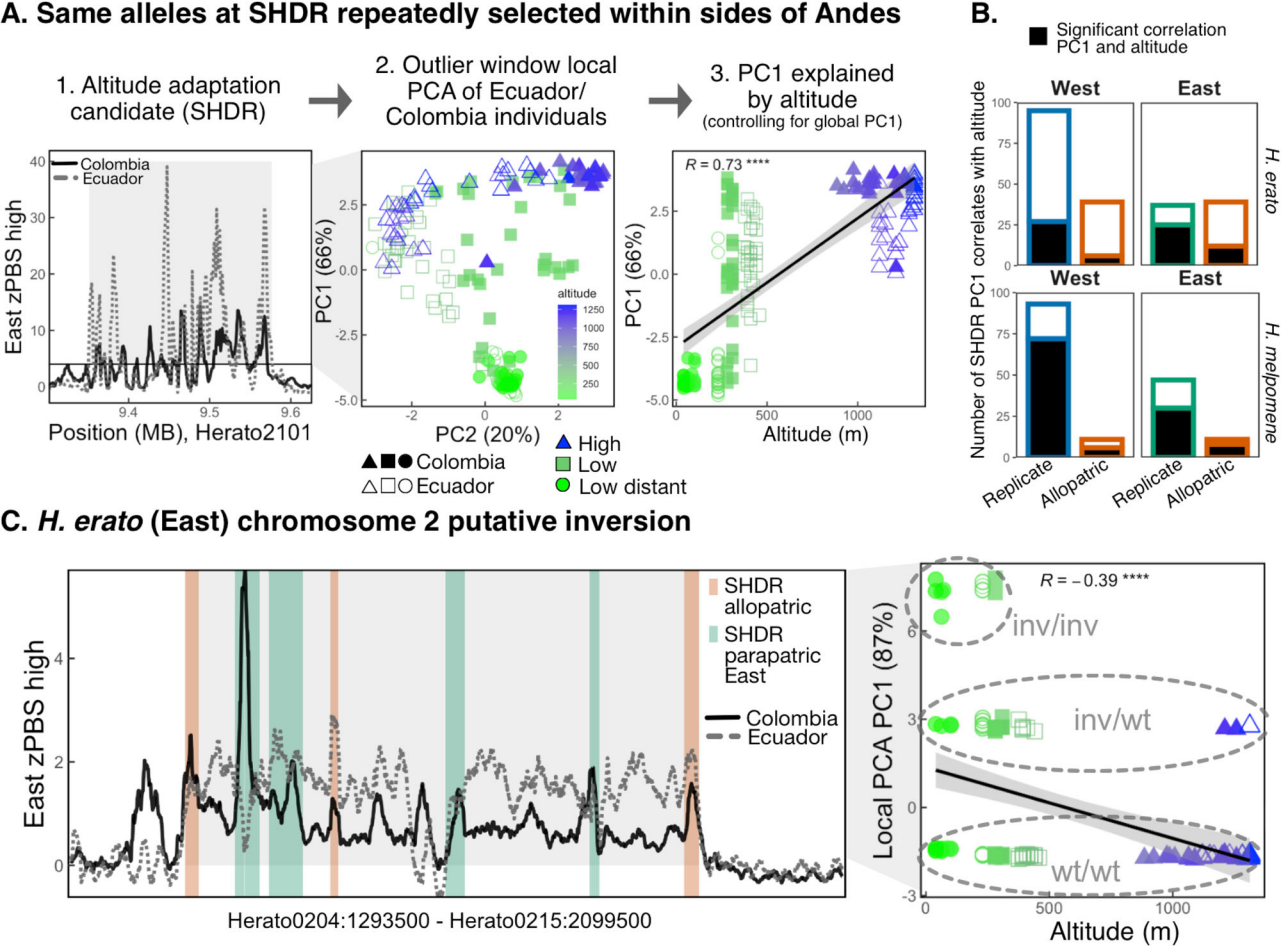
Allele sharing SHDRs and large putative inversion in chromosome 2 of *H. erato* Eastern transects. **A** Example analysis to test whether same alleles underlie SHDRs (here depicted *H. erato* Eastern SHDR #77). First, outlier windows (zPBS > 4) in either the Eastern Colombia (solid black line) or the Eastern Ecuadorian (dotted grey line) transect are selected (grey panel), and a local PCA with those sites is performed. Then we test whether PC1, the axis explaining most of the variation, is significantly explained by the altitude at which individuals were collected, while controlling for the global PC1 (i.e., neutral population structure). Each point represents an individual, their shape represents transect of origin (Colombia filled, Ecuador empty symbols) and their colour the altitude (m) at which individuals were collected. The solid line represents the best fit of a linear model, with the shaded area showing a confidence bands at 1 standard error and the Pearson correlation coefficient is shown (R, P < 2.2 e^−16^). **B** Number of SHDRs where altitude is a significant predictor of local PCA axis 1. **C** zPBS highland differentiation in Eastern Colombia (solid black line) and Ecuadorian (dotted grey line) transects across a 6.5 Mbp region of *H. erato* chromosome 2. zPBS lines are regionally smoothed with rolling means of 200 windows, thus some individual outlier windows are higher in value (Fig. 3). SHDRs are shown as vertical segments, coloured by whether they represent SHDRs within Eastern transects (green) or allopatric shared across all transects (red, SHDRs shown in Fig. 3). The solid line represents the best fit of a linear model, with the shaded area showing a confidence bands at 1 standard error. The Pearson correlation coefficient between the putative inversion local PCA PC1 and altitude is shown (****P < 1.8 e^−7^). Homokaryotes for the wildtype arrangement are named wt/wt, heterokaryotes are inv/wt and inversion homokaryotes are labelled inv/inv. The most common arrangement clustered with the Western transects of *H. erato* and outgroups of other species in a neighbour-joining tree, and thus was considered the most likely, non-inverted haplotype (wt/wt) (Supplementary Fig. 12).

High altitude differentiation at the same locus could be driven by the same or different haplotypes under selection. For instance, different *de-novo* mutations at one locus were recently found to confer parallel adaptation to toxic soils in *Arabidopsis*, although most parallel regions were sourced from a common pool of standing alleles^75^. To test whether our candidate regions shared the same haplotypes, we performed local Principal Component Analyses (PCA) with outlier windows of each SHDR (Fig. 5A). While ‘global’ PCAs tend to show relatedness between individuals due to geographic structure or partial reproductive isolation, local PCAs of smaller genomic regions can highlight divergent haplotypes due to, for instance, structural variation or positive selection leading to similar haplotypes in adapted individuals^76,77^. Here we assessed whether genetic variation across individuals at SHDRs (local PCA PC1) could be significantly explained by altitude while accounting for genome-wide (‘global’) structuring (Fig. 5A) to test for evidence for shared allelic basis for altitude adaptation.

PCAs in each SHDR were performed with individuals from all altitudes in transects connected via gene flow (replicate Colombia/Ecuador transects). Local PCAs at SHDRs often showed individual clustering that differed from the neutral geographic expectations (whole-genome ‘global’ PCAs that included Western or Eastern transects, Supplementary Fig. 1), and the first axes of variation tended to explain a much larger proportion of the variation observed (PC1 explained 55% ± 20 SD compared to 19% in global PCAs, on average; Supplementary Table 3). Out of the four genome-wide PCAs including individuals from replicate transects within sides of the Andes, altitude only explained clustering along PC1 in the Eastern *H. erato* transects (Supplementary Table 3, Supplementary Fig. 1). This can be explained by a different highland colour-pattern subspecies in Colombia, *H. e. dignus*, reducing gene flow across the genome (Fig. 2). In contrast, we found that a large proportion of local SHDR PCAs had PC1s that correlated significantly with individual altitude (*P* < 0.05, *H. erato*: East = 48%, *H. melpomene*: West = 74%, East = 66%, Supplementary Table 3), except in *H. erato* West where only 25% did (Fig. 5B, Supplementary Figs. 8–11). Altitude explained, on average, 12% of the variation in local PC1 while controlling for the global PC1 (altitude partial R^2^, *H. erato*: West = 0.05, East = 0.16, *H. melpomene*: West = 0.10, East = 0.15, Supplementary Table 3).

Local PCAs can also highlight divergent haplotypes, putatively associated with inversions, by clustering individuals that possess homozygous or heterozygous haplotypes in those regions^78,79^. We found several *H. erato* SHDRs within a high differentiation block in chromosome 2 (6.5 mega base pairs, Mbp, Fig. 5C), ~0.75 Mbp downstream from a recently identified inversion exclusively present in lowland individuals of southern Ecuador^64^. We performed an additional local PCA across the large putative inversion and found a three-cluster pattern, consistent with the presence of the three inversion genotypes (Fig. 5C), and a neighbour-joining tree with outgroups supported its appearance in the Eastern lowlands (Supplementary Fig. 12). Local PCAs of SHDRs within the inversion region correlated more strongly with altitude than putatively inverted-only haplotypes, indicating that adaptive loci remain differentiated in the highlands and may pre-date the inversion event in the lowlands (Supplementary Fig. 8). This is consistent with a model in which the inversion could enhance local adaptation by reducing gene flow between elevations at pre-existing locally-adapted alleles^80^.

Overall, the majority of SHDRs involve the same alleles across transects connected via gene flow. Those SHDRs that do not correlate with altitude in local PCAs could represent false positives where there is no selection associated with altitude. However, they may also represent cases where the original beneficial mutation recombined onto distinct haplotypes that spread in different regions; cases where different de-novo mutations arose at the same or nearby loci in different genetic backgrounds in different regions; or where beneficial alleles entered the population through independent introgression events. An alternative hypothesis is that the observed parallelism is partly driven by different selective pressures on each cline, and that these peaks represent ‘evolutionary hotspots’ — regions particularly prone to driving phenotypic evolution across lineages^5^. This seems less likely given that the same haplotypes underlie parallel SHDRs and there is low parallelism between the two species. Nevertheless, putative intra-lineage hotspots could be investigated by assessing mutational biases and chromosomal instability in these regions, as well as disentangling the individual selective factors at play.

The large putative inversion found exclusively in the lowlands may represent a case of structural variation facilitating adaptation in the highlands^80^. Recent studies on environmental adaptation in seaweed flies and sunflowers, among others, have demonstrated a key role for inversions in maintaining adaptive alleles together and facilitating the evolution of locally adapted ecotypes^76,77^. By studying individual clustering across differentiated loci we have shown that the same alleles often drive parallelisms involved in local adaptation. We thus next turned to identifying the source of the genetic variation causing molecular parallelism across populations.

### The source of parallelism: standing variation and adaptive introgression with high-altitude relatives

The presence of the same putatively adaptive haplotypes on several transects could either reflect: (i) standing variation being repeatedly selected in the highlands or shared via intraspecific gene flow, or (ii) recruitment of adaptations from other high-altitude adapted species through introgression. With five high-depth individuals per population of *H. erato* and *H. melpomene*, and 116 additional whole-genomes of high-altitude specialist species and outgroups, we tested for signatures of shared standing variation within species and of adaptive introgression between species in SHDRs.

**Fig. 6:**
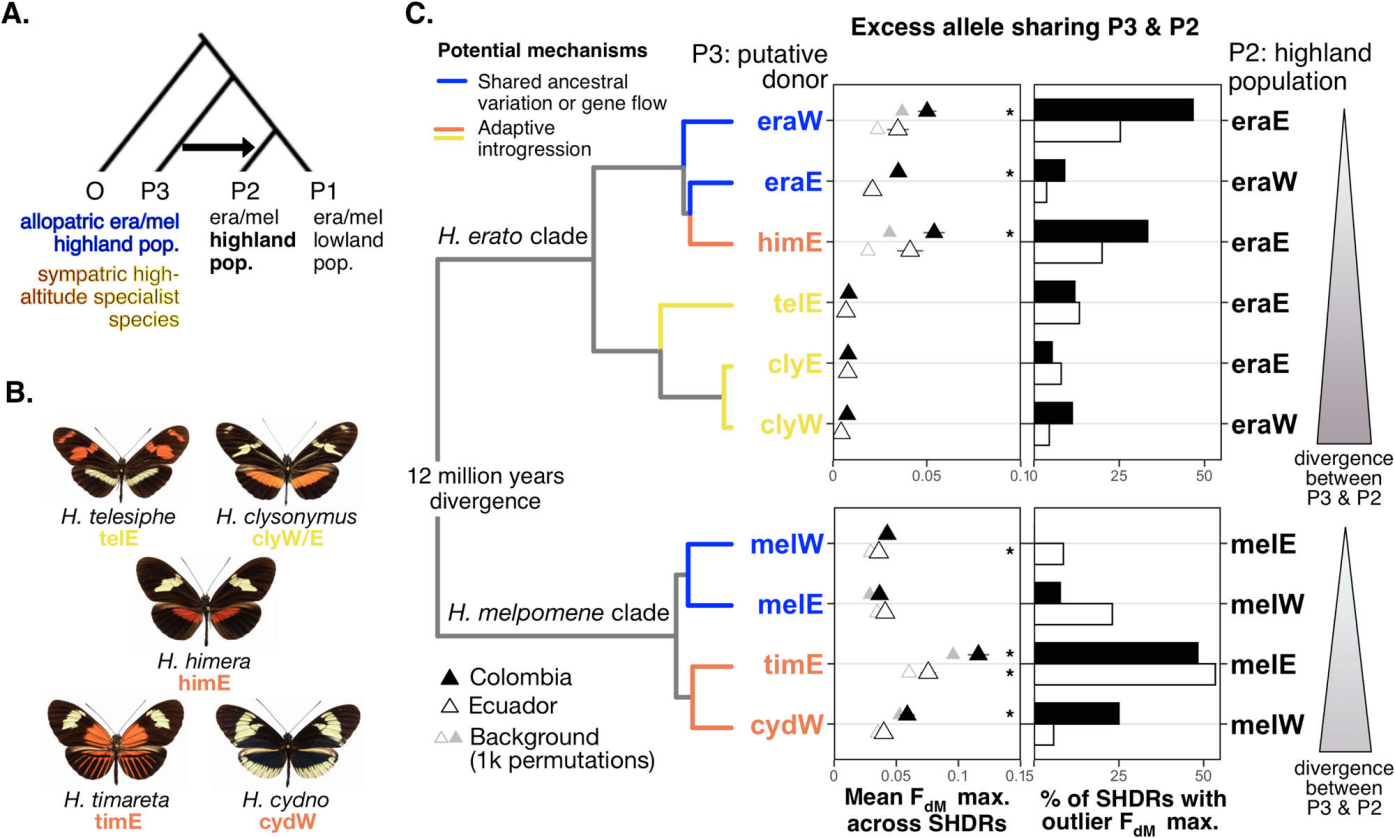
Many SHDRs were sourced from standing variation and putative adaptive introgression from highland-specialist species. **A** Tree used to estimate F_dM_ values per 50kb window across the genome in each comparison, which when positive represents excess allele sharing between P2, a highland *H. erato* or *H. melpomene* population, and P3, an allopatric highland population of the same species or a sympatric high-altitude specialist species, compared to a lowland population (P2). Colours of P3 populations or species indicate the potential mechanism driving the excess allele sharing, either intraspecific shared ancestral standing variation (blue) or adaptive introgression from closely (orange) or distantly related (yellow) high-altitude specialist species. **B** Putative donor (P3) high-altitude specialist species. **C** Excess allele sharing at SHDRs between P3 (putative highland donors, left y axes) and P2 (putative highland recipients, right y axes) across the *H. erato* (top) or *H. melpomene* (bottom) comparisons (phylogeny from Kozak et al.)^1,38^. Left panel shows mean maximum F_dM_ ( ± S.E.) across SHDRs (western SHDRs if the putative recipient was on the West of the Andes, and vice versa) of the Colombian (solid triangles) and Ecuadorian (unfilled triangles) transects. Background mean maximum F_dM_ values were obtained from 1000 block permutations across the genome and shown in grey. Stars represent comparisons where distribution of maximum F_dM_ (excess allele sharing with the highlands) was significantly higher than absolute minimum F_dM_ (excess allele sharing with the lowlands) distribution across all SHDRs (two-sample Kolmogorov-Smirnov tests *P* < 0.05; significant *P*-values from top to bottom: 0.00046, 1.6 e^−5^, 2.0 e^−7^, 0.00031, 0.007, 2.0^−10^, 2.6 e^−8^; Supplementary Figs. 15, 16). Right panels show percentage of SHDRs with evidence of excess allele sharing between P2 and P3, considered significant if they had outlier maximum F_dM_ (> 90th percentile of absolute minimum F_dM_ across all SHDRs). Abbreviations not depicted: era *H. erato*, mel *H. melpomene*. Source data are provided in the Source Data file.

To test for excess allele sharing at SHDR, we calculated the F_dM_ statistic in 50 kb windows across the genome^81,82^. For each test, we used a tree with four populations (((P1, P2), P3), O), where P1/P2 reflect the lowland and highland populations, respectively, and P3 is an allopatric high-altitude population of the same species or a sympatric high-altitude specialist species (Fig. 6A). Positive F_dM_ values indicate excess allele sharing between P3 and P2 (i.e., between non-sister high-altitude lineages), and negative values indicate excess allele sharing between P3 and P1 (i.e., between non-sister high- and low-altitude lineages, Fig. 6A). We then tested if SHDRs are enriched for outlier positive F_dM_ (i.e., excess allele sharing with the highlands), using the distribution of absolute negative F_dM_ across SHDRs as a null (see Methods for details). This specifically tests whether genomic regions that are differentiated in high-altitude populations (SHDRs) are systematically enriched for alleles shared with allopatric high-altitude populations of the same species or with sympatric specialist high-altitude species.

We first assessed intraspecific allele sharing between allopatric highland populations on opposite sides of the Andes, which split ~0.9 million years ago (Mya) and ~1.7 Mya in *H. erato* and *H. melpomene*, respectively^36,37^. Signatures of allele sharing likely represent shared ancestral standing variation that pre-dates the Andean split (Fig. 1B), but we cannot rule out gene flow via distant contact zones in the north and south edges of the Andes or periods of secondary contact in the past^37^. Nearly half of *H. erato* Eastern SHDRs had outlier excess allele sharing with the Western highlands in Colombia, whereas only 9% of Western SHDRs did (Fig. 6B, Supplementary Fig. 15). Both comparisons resulted in a significant enrichment of excess allele sharing between allopatric highland populations compared to sharing with the lowlands across all SHDRs (Kolmogorov-Smirnov tests *P* < 0.05 as stars in Fig. 6B, Supplementary Fig. 15). *H. melpomene* only showed significant enrichment of excess allele sharing between allopatric highland populations of Ecuador in Eastern SHDRs, and the percentage of SHDRs with excess allele sharing was generally lower than in *H. erato* (Fig. 6B). Overall, shared standing variation has been an important mechanism facilitating molecular parallelism in *H. erato*, in which trans-Andean populations share a more recent common ancestor.

We then explored allele sharing between highland populations of *H. erato* and *H. melpomene* and five sympatric high-altitude specialist relatives (Fig. 6C). These highland species are known to readily or occasionally hybridise with *H. erato* or *H. melpomene*^37,83–85^. To quantify genome-wide evidence of allele sharing we first computed f-branch statistics, which test for gene flow between branches of a phylogeny^86^. As expected, we found evidence of excess allele sharing between all relevant pairs (details in Supplementary Note 4, Supplementary Figs. 13, 14)^87^. Excess allele sharing at SHDR between highland populations and sympatric high-altitude specialist species likely represent cases of adaptive introgression. Generally, the more closely related putative donor and recipient species were, the higher proportion of SHDRs that showed excess allele sharing (Fig. 6B, Supplementary Fig. 16). For instance, levels of allele sharing in SHDRs were much higher between highland *H. erato* and the closely related *H. himera* than with the distantly related *H. telesiphe* (Fig. 6B). Context-dependant mutational effects (epistasis) in divergent genetic backgrounds could explain the lower levels of excess allele sharing between distantly related species^21^, as shown for parallel adaptation to altitude in birds^88^. Interestingly, in Eastern *H. melpomene*, SHDR sharing with a closely related sympatric species, *H. timareta*, was more prevalent in SHDR than shared variation with allopatric highland populations of its own species (Supplementary Fig. 16D). Admixture between adjacent *H. melpomene* and *H. timareta* populations is well-documented, with strongly selected colour pattern loci having been shared across the species barrier^84,85,89^.

Levels of putative adaptive introgression at SHDRs were also high between *H. himera* and highland *H. erato*, and significantly enriched across SHDRs in Colombia (Fig. 6B). *H. himera* is a closely related species that split from within the Eastern *H. erato* clade 215,000−527,000 years ago^37^, with pre-mating isolation and a range of divergent life-history phenotypes adapted to the highland dry forests it inhabits^90–92^. Admixture is predominantly from *H. himera* into *H. erato*^37^, supporting our hypothesis that the high levels of excess allele sharing at SHDRs between the two may represent cases of adaptive introgression into *H. erato*. Our study is the first to show that putative adaptive alleles other than colour pattern loci have been shared between high-altitude specialist species and *H. erato* and *H. melpomene*, potentially facilitating their adaptation to montane habitats.

The 6.5 Mbp putative inversion detected in chromosome 2 of Eastern *H. erato* individuals showed high levels of allele sharing between highland *H. erato* populations and *H. himera*, whereas there was no excess allele sharing with either the highlands or the lowlands when the putative donor species was a distantly related species (Supplementary Fig. 17). A neighbour-joining tree of this region revealed that lowland distant individuals that clustered in the local PCA formed a monophyletic group divergent from all other *H. erato* populations, including allopatric populations in the west of the Andes (Supplementary Fig. 12). This suggests that the inversion may have arisen anciently, prior to the western and eastern Andean split of *H. erato*. Its maintenance in the lowland populations may protect locally adaptive alleles from maladaptive migration load and/or promote the accumulation of novel, locally adaptive mutations^77^. Furthermore, its absence in the highlands allows for ongoing gene flow between highland *H. erato* populations and the closely related highland specialist *H. himera*. Supergene evolution in another species of this genus, *H. numata*, has been linked to the introgression of a chromosomal inversion^93^, highlighting the role of structural variation and hybridisation in providing novel genetic architectures that can promote adaptation. Future work could investigate the potential role of this inversion in maintaining locally beneficial allele clusters and their associated adaptive phenotypes.

By studying recently and anciently diverged populations at different altitudes within and across sides of the Andes of two species we have uncovered (i) strong signatures of high-altitude differentiation in narrow regions across the genome, consistent with positive selection, (ii) high levels of molecular parallelism between transects of the same species but no sharing across species, and (iii) an important role of standing variation and adaptive introgression from high-altitude specialist species in adaptation to these montane environments. The overall lack of molecular parallelism across species points towards genetic redundancy of polygenic evolution that allows different combinations of alleles to confer adaptation to the same environments or may reflect variation in the importance of specific selection pressures across species^28^. The evolutionary success of *H. erato* and *H. melpomene* in inhabiting a wide range of altitudes has likely been facilitated by abundant genetic diversity, as well as by intra- and interspecific gene flow allowing for the sharing of pre-existing adaptive alleles. Together, our study highlights the value of extensive replication across space and large whole-genome datasets for understanding the molecular underpinnings of local adaptation in the wild. Both standing genetic variation and recent hybridization can supply the selection targets required for adaptation to the environment, which emphasizes the importance of preserving gene flow and connectivity between populations if organisms are to adapt to everchanging environmental pressures.

## Methods

Our study did not require ethical approvals. In Colombia, field collections were conducted under permit no. 530 issued by the Autoridad Nacional de 539 Licencias Ambientales of Colombia (ANLA). In Ecuador, collections during November-December 2011, and September-October 2012, were done under permit 0033-FAU-MAE-DPO-PNY and exported under permits 001-FAU-MAE-DPO-PNY and 006-EXP-CIEN-FAU-DPO-PNY. Permits were obtained from Parque Nacional Yasuní, Ministerio Del Ambiente, La Dirección Provincial de Orellana. Collections in Ecuador during 2017-2019 were conducted under the permit provided by the Ministerio del Ambiente, Ecuador (MAE-DNB-CM-2017-0058).

### Study system and wild butterfly collection

*H. erato* and *H. melpomene* can be found across most of the Neotropics and have Müllerian aposematic mimicry to advertise their toxicity to predators, thus share colour pattern when inhabiting the same areas^94^. They can be found continuously coexisting across altitudinal transects ranging from sea level up to 1600 m along the Andean mountains, and *H. melpomene* can be found across elevations up to 1800 m. Butterflies were collected from 111 different locations, which we grouped into 30 populations, corresponding to four transects Colombia West/East, and Ecuador West/East (Supplementary Table 1). In each transect populations were either in the highlands (altitude mean = 1235 m), lowlands (altitude mean = 364 m), or distant lowlands (altitude mean = 95 m) to control for genetic drift due to isolation by distance (Fig. 2A). The Andes acts as a barrier to gene flow, as elevations in these latitudes are too high for butterflies to fly across and have been for at least 8 million years^95^, which pre-dates the expansion of both species across these latitudes^36,37^. Microclimatic variation across the Ecuadorian altitudinal transects here studied were found to be remarkably similar between Eastern and Western transects^33^, making them ideal for the study of repeated adaptation. All but one of these transects (Colombia East) had the same subspecies, i.e., geographic colour morph, in the three altitudes, to avoid differentiation due to highly divergent colour pattern loci (Fig. 2A). Detached wings were stored in glassine envelopes and bodies in EtOH (96%) vials. We additionally collected high-altitude specialist relatives of *H. erato* and *H. melpomene* that have potential for admixture between them. The *H. erato* relatives were *H. himera* and *H. telesiphe* from the Eastern Andes, and *H. clysonymus* which is found on both sides of the Andes. The *H. melpomene* relatives were *H. timareta* and *H. cydno*, from the Eastern and Western Andes, respectively. More distantly related outgroups were also sampled, *H. eleuchia* and *H. hecale* for *H. erato* and *H. melpomene*, respectively.

### Whole-genome sequencing

Whole genome sequence data from 518 individuals was analysed in this study, 444 were sequenced for this study, while the rest were obtained from published studies (*n* = 74). Of the individuals sequenced for this study 365 were sequenced at low-medium depth with BGI (~6X), and 79 were sequenced at high depth with Novogene (~18X-30X), at least 5 per population. For the high-altitude specialist species dataset and outgroup species, we obtained high-depth whole genome sequencing data for 116 individuals, 63 of which were sequenced for this study at ~20X depth with BGI. A full list of individuals, localities, and accession numbers can be found in the Supplementary Data 1. Individuals with *H. melpomene malleti* phenotypes (Fig. 1) were genotyped with PCR amplification with the primer Jerry followed by a restriction digest, following^56^, to identify cryptic individuals of the species *H. timareta ssp. nov*., which are indistinguishable phenotypically from *H. m. malleti*. We extracted DNA with QIAGEN DNeasy Blood and Tissue extraction kits, including RNA removal, and confirmed DNA integrity and concentration (minimum of 10 ng/μL) using Qubit. DNA samples were stored at −20 °C until library preparation. For the individuals that were sequenced with low-medium depth, a secondary purification was performed with magnetic SpeedBeads^TM^ (Sigma) and we prepared Nextera DNA libraries (Illumina, Inc.) with purified Tn5 transposase^96^. PCR extension with an i7-index primer (N701–N783) and the N501 i5-index primer was performed to barcode the samples. Library purification and size selection was done using magnetic SpeedBeads^TM^ (Sigma). We confirmed adaptor lengths through TapeStation High sensitivity T1000 (Agilent Technologies, CA, USA) and gel electrophoresis. Pooled libraries were sequenced by The Beijing Genomics Institute (China) using HiSeq X Ten (Illumina). Library preparation and sequencing of the high-depth *H. erato* and *H. melpomene* individuals was carried with HiSeq X platform (150 bp paired-end) by Novogene.

### Statistical analyses

All non-genomic analyses were run in R V2.13 (R Development Core Team 2011) and graphics were generated with the package *ggplot2*^97^.

#### Read mapping and genotype calling

We aligned the sequence data of all individuals of the two focal species and their relatives to their corresponding reference genomes, either *H. melpomene* version 2.5^98,99^ or *H. erato demophoon*^58^, obtained from Lepbase^98^, using bwa mem (v 0.7.15^100^). We used samtools (v 1.9^101^) to sort and index the alignment files. Duplicates were removed using the MarkDuplicates program in Picard tools (v 1.92 Broad Institute, 2018^102^). Genome-wide mean sequencing depth was calculated with samtools (v 1.9^101^). Mean sequencing depth was very similar across areas of *H. erato* (mean = 8.93, Supplementary Fig. 18 and was generally higher for *H. melpomene* (mean = 12.3), but more variable, especially in Colombia where many sequences were obtained from published studies (Supplementary Data 1). Most of the analyses described below for *H. erato* and *H. melpomene* were performed with genotype likelihoods in ANGSD and low or variable sequencing depths are thus accounted for^103^.

However, for our phylogenetic datasets combining our samples with other species for phylogenetic tree reconstruction and tests of admixture, we restricted the *H. erato* and *H. melpomene* samples to the five individuals per population with high sequencing depth. We mapped the high-altitude specialist relatives and outgroups of *H. erato* and *H. melpomene* to the respective reference genomes as explained above (Supplementary Data 1). We used a genotype calling approach with GATK v. 3.7^104^ to obtain a vcf file each for the *H. erato* and *H. melpomene* clade. Genotypes were called with HaplotypeCaller for each individual and variants were then called with GenotypeGVCFs across all individuals combined. The vcf files were filtered with vcftools v. 0.1.15^105^ to remove genotypes with less than 3 reads, monomorphic sites, multi-allelic sites, insertions and deletions (indels), and sites with more than 50% missing data.

#### Isolation by distance and Isolation by environment

To study Isolation by Distance (IBD) and Isolation by Environment (IBE), we first calculated all pairwise genetic differentiation (Fst) between all populations on each side of the Andes that had at least 5 individuals each, i.e., *H. erato* west (*n*_population_ = 7), *H. erato* east (*n*_population_ = 11), *H. melpomene* west (*n*_population_ = 7), *H. melpomene* east (*n*_population_ = 9), hereafter side-species replicate (Supplementary Table 1). We calculated pairwise population genetic distance with the function calculate.all.pairwise.Fst() from the R package BEDASSLE^39^. This requires a matrix of allele count data, with populations as rows and number of biallelic unlinked loci sampled as columns, which we obtained with ANGSD and custom scripts. First, we obtained a list of highly polymorphic SNPs per side-species replicate, by (i) heavily filtering 10 random individuals and obtaining minor allele frequencies (-doMaf 1) by forcing the major allele to match the reference state, so that it is the same across all populations (-doMajorMinor 4) (ii) extracting the sites and the major/minor allele frequencies, (iii) creating an indexed sites file (angsd sites index), (iv) subsetting so that sites are at least 2 kb apart, to prune for linkage disequilibrium. This list of sites and regions was then used to obtain minor/major allele frequency counts with all individuals per population and forcing the major/minor allele to match the ones given by the sites file (-doMajorMinor 3). From the resulting allele frequencies per population, we calculated allele counts, by multiplying by the number of individuals per population and the number of chromosomes samples (2, diploid). We obtained the required allele count matrix by concatenating all populations per side-species replicate, and only keeping loci with allele counts for all populations.

Geographical distance between populations measured as a straight line through the landscape is not biologically representative of organisms moving through space. To account for topographic complexity, we obtained topographic least cost paths with the R package *topodistance*. With historical records from the Earthcape database^106^, we created a binary habitat suitability raster based on the altitudinal range of each species, so that least cost paths between populations never included elevations that these butterflies do not inhabit. Then we used the function topoLCP() to get the least cost path distance between populations (Supplementary Fig. 3^107^), and use this distance as a proxy of isolation by distance between populations.

#### Differentiation and selection statistics

To search for signatures of local adaptation to montane habitats we use a measure of lineage-specific differentiation, population branch statistics (PBS)^44^. PBS is a summary statistic based on pairwise genetic differentiation (Fst) among three populations, two of which are located closely geographically (high, low) and one distant outgroup (low distant). For each population trio (high, low, low distant Fig. 1A), we computed PBS with ANGSD^103^. We first obtained genotype likelihoods to calculate the site-frequency spectrum (SFS) per population. Then, we computed 2D-SFS for each population pair (high-low, high-low.distant, low-low.distant) with the function realSFS. We then used the 2D-SFS as a prior for the joint allele frequency probabilities at each site, which are used to compute per-site pairwise Hudson’s Fst^108^ as interpreted by Bhatia (^109^, realSFS fst index -whichFst 1) among the three populations (Fst_high-low_, Fst_high-low_, Fst_high-low.distant_) and PBS per population (PBS_high_, PBS_low_, PBS_low.distant_). This is achieved by first transforming pairwise Fst values into relative divergence times with Eq. (1):

1 $$T=-{\log }\left(1-{Fst}\right)$$

To then obtain PBS for a given population (here the highlands) with Eq. (2):

2 $${{PBS}}_{{high}}=\frac{{T}_{{high}-{low}}+{T}_{{high}-{low}.{distant}}-{T}_{{low}-{low}.{distant}}}{2}$$

This quantifies the magnitude of allele frequency change in the highland lineage since its divergence from the lowland and lowland distant populations. Regions of the genome with large PBS values represent loci that have undergone population-specific sequence differentiation consistent with positive selection. We computed weighted Fst and PBS averages per 5 kb window size and 1 kb steps (realSFS fst stats2), with the same window centres across datasets (-type 0). For transects where only two out of three populations had any individuals (Colombia west for *H. erato* and Ecuador west for *H. melpomene* Fig. 1A), we computed pairwise Fst only. Finally, we normalized PBS and Fst window values with z-scores, i.e. number of standard deviations from the mean, so that divergence is comparable across transects.

#### High Differentiation Region parallelism between replicates

To measure genetic parallelisms in adaptation to the highlands, we quantified overlap of outlier windows and adjacent regions, i.e. high differentiation regions (HDR), across replicate and allopatric transects and species. We considered outlier windows to be those with values above 4 standard deviations from their mean (zPBS_high_ > 4, zFst > 4; following^51^). We then expanded outlier window positions 50 kb upstream and 50 kb downstream from the window centre (HDR) and checked for overlaps with other transects, either within or across sides of the Andes for each species, to highlight parallelism when visualizing patterns of genome-wide divergence. High-differentiation regions with any overlap with other transects are termed shared HDRs (SHDRs). To check for HDR overlaps between species, we mapped the *H. melpomene* windows (starts and end positions) to the *H. erato* reference genome using a chainfile^64^ and the liftover utility (Hinrichs, 2006).

To test whether the level of parallelism observed between transects within and across sides of the Andes was higher than expected by chance, we used the R package *intervals*^110^. We first created outlier window intervals (HDRs), by obtaining the start and end positions of continuous blocks of outlier windows ( ± 50 kb buffers) with the function Intervals() (options Type=”Z”, closed). We obtained the observed proportion of total intervals that overlapped, at any of their positions, with outlier intervals in the other transect within sides of the Andes, or with outlier intervals in both, the other transect within the same side of the Andes and the two transects on the other side of the Andes (allopatric sharing). We then simulated 10,000 randomized distributions of outlier-window intervals across the genome per transect, per species (*n* = 8). In each simulation set, we randomly placed the same number of HDR intervals and of the same size as the observed outlier-window intervals for those transects. With these, we estimated the proportion of simulated intervals that overlapped with observed HDR outlier intervals within and across sides of the Andes, obtaining as a result a null distribution of interval overlap proportions. This approach does not account for the possibility that different parts of the genome may be more readily involved in local adaptation. Finally, to determine whether the level of overlap was significantly greater than expected by chance alone, we performed jackknife block resampling across the genome to estimate the 95% CI for the observed proportion of overlapping intervals and then assessed whether this interval included the expected value, which was computed as the mean of 10,000 random permutations described above.

#### Measures of nucleotide diversity, selective sweeps, and recombination rate

We studied genetic variation within and across populations by deriving three summary statistics from ANGSD thetas estimations^103^, Tajima’s D, which estimates the deviation of a sequence from neutrality, nucleotide diversity (pi, or population mutation rate), and Dxy or absolute divergence, which calculates pairwise differences between sequences of two populations excluding differences between sequences within populations. ANGSD has been found to be an accurate estimator of nucleotide diversity because it includes invariant sites^111^. Firstly, we obtained folded global site-frequency spectra for each population. Then we calculated pairwise nucleotide diversity per site (thetaD, realSFS saf2theta). Finally, we performed sliding window analysis of 5 kb window size and 1 kb steps (thetaStat do_stat) to obtain sum of pairwise differences, Tajima’s D, and total effective number of sites per window. Nucleotide diversity (pi) was obtained by dividing the sum of pairwise differences by the total number of sites per window. For all transects, we calculated the difference in nucleotide diversity per window between highland and lowland distant populations (or low populations if low distant individuals unavailable), which is expected to be negative if a selective sweep led to locally reduced diversity in highland populations. Absolute divergence (D_xy_,^112^) between high (population A) and low/low distant (population B) populations was estimated by additionally obtaining pairwise nucleotide diversity per site (thetaD) for all individuals pooled from populations A and B (population AB), and then per-site D_xy_ obtained with Eq. (3):

3 $${{Dxy}}_{{site}}=\frac{{thetaAB}\times \left(\genfrac{}{}{0ex}{}{{nAB}}{2}\right)-\,{thetaA}\times \left(\genfrac{}{}{0ex}{}{{nA}}{2}\right)\,-\,{thetaB}\times \left(\genfrac{}{}{0ex}{}{{nB}}{2}\right)}{{nA}\,\times \,{nB}}$$

With n being the number of individuals per population (A, B, or pooled AB) and thetaD obtained from realSFS saf2theta. Mean D_xy_ was estimated for the same 5 kb windows with 1 kb steps. Finally, we obtained recombination rate for 50 kb windows along the genome from the mean population recombination rate (*ρ* = 4*N*_e_*r*; *r* = probability of recombination per generation per bp) estimated for 13 *H. erato* populations from across the range in a recent study^113^ and for 100 kb windows of four *H. melpomene* populations^71^. Note that the *H. erato* genome is larger than *H. melpomene* (383 Mb and 275 Mb, respectively), hence the difference in window sizes.

#### Testing for significance of selection statistics in SHDRs

To test for positive selection we assessed Tajima’s D, difference in nucleotide diversity across elevations Δπ (π_high_− π_low_), and absolute divergence (D_xy_) within SHDRs and compared values to simulated distributions. We used the same permutation approach described for assessing HDR parallelism, by randomly placing the same number of intervals and of the same size as the observed HDRs for each transect 10000 times. We then obtained minimum Tajima’s D and Δπ and maximum D_xy_ within each simulated SHDR and permutation, and only considered a SHDR to be an outlier for a given selection statistic if the observed maximum (Tajima’s D, Δπ) or minimum (D_xy_) value was above the 90th or below the 10th percentile of simulated values. Number of outlier selection statistics per SHDR was tallied and compared across replicate or allopatric sharing in each species.

#### Global and local PCAs

To assess neutral genetic variation between individuals and populations, we performed principal component analysis (PCA) in the eastern and western transects separately for each species, i.e., two transects per PCA (Fig. 1A). We first obtained a random subsample of 10% of windows that did not have high differentiation across populations, i.e., with zPBS/zFst < 4, and then pruned for linked sites by only retaining 1 site for every 10 kb, yielding 14995 and 8293 sites for *H. erato* and *H. melpomene*, respectively. We used the program ANGSD (v 0.933^103^) to obtain genotype likelihoods in beagle format (-doGlf 2) for all individuals. In *H. erato*, we excluded chromosome 2 as it contains a large inversion which could distort the neutral differentiation landscape. Genotype likelihoods were used as input for PCAngsd^114^, which incorporates genotype uncertainty from genotype likelihoods to obtain a covariance matrix across all individuals.

To assess whether the same haplotypes were involved in adaptation to altitude across replicate transects (within sides of the Andes), we performed local PCAs with outlier windows (zPBS/zFst > 4, i.e. >4 standard deviations from the mean) of each SHDR (total = 370 local PCAs). All individuals from replicate transects were included, leading to local PCAs for all Western and Eastern SHDRs of each species that included Colombia and Ecuador samples. We obtained genotype likelihoods in beagle format (-doGlf 2) as input for PCAngsd, similarly to the population structure analysis. We then assessed whether altitude was a significant predictor of individual clustering in each SHDR by building a linear model where local PCA PC1 was the response variable, and altitude and global (genome wide) PCA PC1 the predictors, to account for population structure. We considered that individuals in each replicate transect had the same haplotypes in SHDRs if altitude was a significant predictor of local PCA PC1, while controlling for global PC1 (Fig. 5). We additionally obtained the overall variation explained by the fitted linear models (R^2^) for each SHDR local PCA and the relative contributions of each explanatory variable (altitude and global PCA PC1, partial R^2^), estimated with the package *relaimpo*^115^.

#### Measures of excess allele sharing

We used *ABBA-BABA*-related statistics to examine patterns of allele sharing between closely or distantly related high-altitude species and our study *H. erato* and *H. melpomene* populations. These statistics test for an excess of shared derived variation between lineages to distinguish gene flow or ancestral population structure from the incomplete lineage sorting (ILS) that can occur during a simple tree-like branching process. To examine genome-wide patterns of excess allele sharing between populations and species, we obtained F branch statistics implemented with the package *Dsuite*^86^. Fbranch summarises and visualises patterns of excess allele sharing across phylogenetic datasets. We performed linkage-pruning to obtain a genome-wide average of excess allele sharing. Using a custom script (https://github.com/joanam/scripts/blob/master/ldPruning.sh) we removed sites above an LD-threshold of R^2^ > 0.1 with plink v. 1.07^116^. To reconstruct the backbone phylogeny for excess allele sharing tests, we extracted for each population or species the individual with highest sequencing depth from the vcf file using vcftools v. 0.1.15^105^. The vcf file was then converted to phylip with a custom script (https://github.com/joanam/scripts/blob/master/vcf2phylip.py). We reconstructed the phylogeny of the melpomene and erato clade separately with RAxML v. 8.2.9 using the GTRGAMMA model^117^. Using this backbone tree, we used the LD-pruned vcf files of all melpomene/erato clade individuals to computed f statistics (tests of excess allele sharing) across all possible sets of three populations or species with Dsuite Dtrios. Next, we summarized these statistics with Dsuite Fbranch^118^. The extent of gene flow in the eastern Andes between *H. melpomene* and *H. timareta* can skew genome-wide trees. Thus, to assess the levels of gene flow, we constrained the *H. melpomene* clade to be monophyletic following the species tree. In order to remove spurious signatures of excess allele sharing that are not significant, we set Fbranch values to 0 if the z-score was greater than 3 with a custom script (https://github.com/joanam/scripts/blob/master/removeNonsignDsuite.r). Lastly, we plotted the Fbranch statistics along the phylogeny with dtools.py of the Dsuite package.

To test for adaptive introgression from high-altitude specialist species into *H. erato* and *H. melpomene* highland populations, we computed *f*_dM_, a statistic of excess allele sharing suitable for small genomic regions. This test is based on a set of four populations, including two sister taxa (P1 & P2), a close relative (P3) that may have admixed with one of these sister taxa and an outgroup (O). Here, the sister taxa represent the *H. erato* or *H. melpomene* lowland (P1) and highland populations (P2), whereas P3 represents a highland specialist species that may have contributed beneficial gene variants to the *H. erato* and *H. melpomene* highland populations. f_dM_ quantifies gene flow between P3 and P2 or between P3 and P1. In addition, we ran these tests with the allopatric erato/melpomene populations as P3, to test if at SHDRs, the same haplotypes are found in high-altitude populations on both sides of the Andes, potentially due to parallel selection on the same haplotypes. We estimated f_dM_ for non-overlapping 50 kb windows across the genome with the ABBABABAwindows.py script by (Martin et al. 2014) from https://github.com/simonhmartin/genomics_general. We considered individual SHDRs as f_dM_ outliers if their observed maximum f_dM_ value was > 90th percentile of the absolute minimum f_dM_ values across all SHDRs. Additionally, we tested for overall enrichment of excess allele sharing between P3 and P2 (i.e., with the highlands) across all SHDRs, by testing with a two-sample Kolmogorov-Smirnov tests if the distribution of maximum f_dM_ values across all SHDRs (i.e., allele sharing with the highlands, P2) was significantly higher than the absolute minimum f_dM_ values across all SHDRs (i.e. allele sharing with the lowlands, P1). This was repeated for each Colombia/Ecuadorian clines with their respective potential donors (P3).

### Reporting summary

Further information on research design is available in the Nature Research Reporting Summary linked to this article.

## Supplementary information

Supplementary Information

Description of Additional Supplementary Files

Supplementary Data 1

Reporting Summary

## Data Availability

The sequence data generated in this study have been deposited in the European Nucleotide Archive database under accession code PRJEB35570 or elsewhere on ENA if obtained from previous studies, as specified in Supplementary Data 1 for each individual. Reference genomes for *H. erato* and *H. melpomene* were taken from Lepbase v. 4^98^ (*H. erato demophoon*^58^ and *H. melpomene* version 2.5^99^, respectively). Source data are provided with this paper in the Source Data file and in the relevant public repository published in Zenodo (10.5281/zenodo.6836103)^119^. All records associated to the individuals used for this study are available in the *Heliconius* Earthcape database (https://heliconius.ecdb.io/^106^). Source data are provided with this paper.

## Source data

Source Data

## Code availability

Scripts and pipelines have been made available in the public repository Zenodo (10.5281/zenodo.6836103)^119^.
