# Supplementary material for: Repeated genetic adaptation to altitude in two tropical butterflies
Source: Nat Commun. 2022 Aug 9;13:4676. doi: 10.1038/s41467-022-32316-x (PMC9363431; doi:10.1038/s41467-022-32316-x)
Supplement: Supplementary file 1 — Supplementary Information [file 41467_2022_32316_MOESM1_ESM.pdf]

Supplementary Notes

Supplementary Note 1. Natural history and biogeography of *Heliconius* butterflies

Heliconius show Müllerian mimicry, whereby aposematic colouration is shared among co-occurring species, to advertise their toxicity to predators[1]. There are currently 48 described *Heliconius* species and they span most of the Neotropics, from northern Argentina to southern parts of the United States[1]. Diversification rates in the *Heliconius* adaptive radiation have increased gradually for the last 11 million years. Intense but constant changes in the environment and topography since the mid-Miocene (e.g. uplift of the Andes) have been hypothesized to create a dynamic arena where *Heliconius* speciation and colonisation of new ecological niches has taken place[2,3]. However, recurrent hybridisation across the phylogeny (at least 26% of extant *Heliconius* species can hybridise[4]) make reconstructing the evolutionary history and biogeography of this clade difficult[5].

Species richness is highest in the eastern foothills of the Andes and the upper Amazon lowland forest, although there is considerable niche partitioning, with some species, such as *H. telesiphe* or *H. hierax*, that only inhabit the highlands (up to 3200 m) while others only inhabit the lowlands[6]. A recent study found that variation in species richness and phylogenetic diversity in this genus was largely explained by altitude[7]. The two co-mimetic species studied here (*H. erato* and *H. melpomene)* diverged early on in the radiation 12 million years ago, but can be found coexisting across most of their range, with continuous populations inhabiting altitudes from sea-level to at least 1600 m. *H. erato* and *H. melpomene* stand out in terms of their broad ranges and abundance (map produced with data from https://heliconius-maps.github.io[6] and Map data ©2019 Google obtained via RgoogleMaps package[8]), making them interesting systems to study local adaptation.

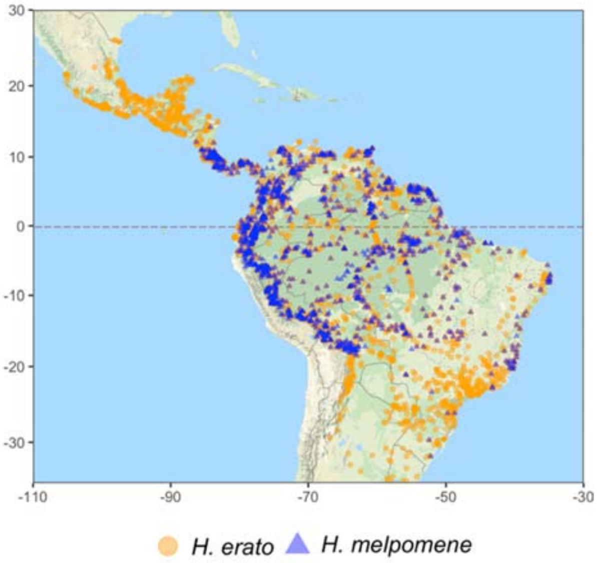

Heliconius feed almost exclusively on the diverse family *Passifloraceae*, or passion vines, which have evolved a range of chemical and physical barriers against herbivores[9,10]. In turn, these butterflies have adapted to, or even co-opted, these defences for their own benefit, detoxifying and sequestering cyanogenic compounds to make them unpalatable to predators. Furthermore, *Heliconius* are the only Lepidoptera known to actively feed on pollen, which gives them a source of protein as adults and has profound effects on their biology and behaviour. Pollen feeding allows for long lifespans of up to nine months and disproportionately large brains for their size[11], enabling them to memorise routes between flowers and follow daily traplines[12,13]. *Heliconius* disperse 2-4km after emergence, but establish a small home ranges to memorise and navigate the complex forest environment as adults[12,14], suggesting high levels of local adaptation. The near-continuous populations of *H. erato* and *H. melpomene* across elevations allow for rampant gene flow, despite little adult dispersal.

Supplementary Note 2. HDR summaries and testing impact of buffer size around outlier windows on parallelism

In the three *H. erato* clines with three populations (PBS), we found an average of 216 HDRs (±47) covering 6.4% of the genome (±1.2%), including upstream and downstream region buffers of 50kb. In the three *H. melpomene* clines with PBS, we found 242 HDRs (±65) covering 10.9% of the genome (±2.5%), on average. The clines for which only two populations were sampled (Fst), had a higher number of HDRs, 400 and 405 HDRs, covering 11.4% and 17.1% of the genome for *H. erato* and *H. melpomene*, respectively.

We tested the effect of changing buffer size on the difference between the observed and simulated proportion of HDRs shared across clines. We found that this difference was generally robust to the size of the buffer, except when increasing the buffer to ±100kb in *H. melpomene*, which led to a larger difference between the observed and simulated values (S.I., Supplementary Figure 18). Thus, we used ±50kb as buffers around outlier windows, which is conservative given that well-studied regulatory regions can be up to 100kb away of positively selected colour pattern loci in these species[15].

Supplementary Note 3. East SHDRs test for additional signatures of selection in published high-quality linked-read dataset

To further validate signatures of selective sweeps in our altitude Shared High Differentiation Regions (SHDRs), we used a recently published linked-read dataset to run a Genome-wide association study with individual altitude as a phenotype and tested for enrichment of selection statistics within SHDRs (Supplementary Figure 5). Four selection statistics were calculated for the highland and lowland populations in Meier *et al*. 2021[16], integrated haplotype score (iHS), nucleotide diversity (Π) across elevations ($\Delta\Pi = \Pi_{high} - \Pi_{low}$), omega ($\omega$), and composite likelihood ratio to detect selective sweeps using sweeD. We additionally performed a genome-wide association study with the same dataset, where the phenotype/response variable was set as the altitude at which individuals had been collected while controlling for sex, wing area, and admixture proportions as covariates. We followed the methods and outlier association region detection pipelines outlined in Montejo-Kovacevich et al. 2021 [17], which used this sequencing dataset to investigate wing shape genomics.

We found that over half of eastern SHDRs of both *H. erato* and melpomene overlapped with at least one selection outlier or GWAS association region of this independent altitudinal re-sequenced cline (Supplementary Figure 5 B, C). As expected, the number of western SHDRs that overlapped with selection outliers was lower, as the dataset from Meier *et al.* 2021 is from individuals collected on a cline on the East of the Andes (Supplementary Figure 5 A). We found that 50% and 15% of eastern parapatric SHDR in *H. erato* and melpomene, respectively, overlapped with strongly altitude-associated regions in the haplotagging GWAS, which make up 0.44% of the genome, whereas only 8% and 11% of parapatric western SHDRs overlapped with GWAS outliers (Supplementary Figure 5 D, E). Allopatric SHDRs showed high levels of overlap with haplotagging-derived selection statistics in all clines (52% ±15 SD, on average)

Supplementary Note 4. Genome-wide evidence of admixture across clades

We assessed genome-wide evidence of ancestral standing variation or admixture by obtaining f-branch statistics from a balanced dataset of five high depth samples per population or species. In the *H. erato* clade, we found strong evidence of genome-wide allele sharing between the highland *H. telesiphe* / *H. clysonymus* clade and the western *H. erato* clade (mean fbranch= 0.034, Supplementary Figure 13). There was excess allele sharing between geographically isolated western and eastern highland populations of *H. erato*, especially in the Colombia western populations (Supplementary Figure 13), indicating the presence of ancestral standing variation as gene flow is absent. Furthermore, the highland species *H. clysonymus*, which is found on both sides of the Andes, had excess allele sharing with all populations of the *H. erato* clade, including the incipient eastern species *H. himera* (mean fbranch= 0.043, Supplementary Figure 13, Fig. 6). In *H. melpomene*, fbranch values were generally higher, probably because of a more closely related set of species and populations, leading to higher admixture (Supplementary Figure 14). Contrary to expectations, in H. melpomene the strongest signatures of genome-wide excess allele sharing corresponded to comparisons with sympatric high-altitude closely related species rather than within-species comparisons between eastern and western clades of the Andes (Supplementary Figure 14). These results support the presence of allele sharing between high-altitude specialist species and the *H. erato* and *H. melpomene* populations under study, which could allow for locally adaptive genomic regions to have been shared via adaptive introgression.

Supplementary Tables

Supplementary Table 1. Population summaries (n=30) corresponding to the points shown in Fig 1A. Nearby localities were considered one population, individual localities listed as they appear on the Earthcape database separated by ";" [18].

Name	Side Andes	Country	Alt.	Localities on Earthcape	# *H. erato*	# H. melp.	latitude	longitude	altitude
flo	East	Colombia	High	Sucre, Florencia; Sucre; Doraditas; Finca C. Piñacué; Quebrada Las Doraditas	4	8	1.795	-75.660	1174
moc	East	Colombia	High	Mocoa-Subundoy site 4; Mocoa-Subundoy site 5	6	0	1.079	-76.716	1235
cam	East	Colombia	High	Campo Cana 02; Campo Cana 01; Campo Cana 06; Campo Cana 03; Campo Cana 04; Campo Cana 07; Campo Cana 09; Campo Cana Mauricio3; Campo Cana 13; Campo Cana 12; Campo Cana 10; Campo Cana; Campo Cana Mauricio1; Campo Cana 11; Campo Cana Mauricio2	14	17	1.214	-76.685	993
bae	East	Ecuador	High	Km 119 Baeza - Lago Agrio; Baeza - Lago Agrio, Rio Salado	0	10	-0.191	-77.697	1360
sum	East	Ecuador	High	Wild Sumaco Hummingbird Trail	0	5	-0.677	-77.599	1468
rev	East	Ecuador	High	Reventador road; Reventador-Sucumbios; San Raphael Falls, Baeza-Lago Agrio,	14	3	-0.030	-77.529	1312
nar	East	Ecuador	High	Reserva Narupa, P10; Reserva Narupa, bridge; Jondachi, Napo, Ecuador; Mina Negra; Finca Narupa, bridge; Finca Narupa; Finca Jose Simbanas P13; Road after y in km24 Tena-Quito	18	7	-0.703	-77.753	1178
bra	West	Colombia	High	Rio Bravo - Calima; Rio Bravo 3; Rio Bravo-Calima; Rio Calima 5km	0	16	3.632	-76.713	1334
que	West	Colombia	High	Queremal; Queremal (Rio San Juan, Vereda El Digua)	21	2	3.504	-76.757	1192
min	West	Ecuador	High	Casa Amarilla, Mindo; Mindo to Finca Birdadvenrure 4; Mindo to Finca Birdadvenrure 1; Mindo to Finca Birdadvenrure 2	11	1	-0.055	-78.785	1245
pac	West	Ecuador	High	Nanegal to Marianita; Pacto- Rio Toali, site 4; Pacto to Paraiso, Rio3; Pacto to Paraiso, Rio1; Pacto- Rio Toali, site PAC4; Pacto to Paraiso, Rio2; Pacto- Rio Toali, site 5; Pacto- Rio Toali, site 2; Pacto- Rio Toali, site 6; Pacto- Rio Toali, site 1; Pacto to Paraiso 1 ; Road to Mashpi (116); Mashpi to Pachijal 2; Road to Mashpi 3 (116); Road to Mashpi 2 (116)	20	28	0.154	-78.760	1097
fll	East	Colombia	Low	Quebrada La Yuca	0	1	1.610	-75.667	340

guz	East	Colombia	Low	Pto Guzman; finca la cimita Guzman	12	7	0.956	-76.409	281
esc	East	Colombia	Low	Finca el Escondite; Via a la Joya-finca el Escondite-vereda san Rafael; Via a la Joya-finca el Escondite-vereda san Rafael	21	2	0.795	-76.585	304
jat	East	Ecuador	Low	Limoncocha - El Carmen 2; Limoncocha - El Carmen 1; San Pedro de Arajuno, Río Arajuno; San Pedro de Arajuno, trail; Jatun Satcha P03; Jatun Satcha, jardin botanico; Puni Bocana, trail; Puni Bocana, lodge; Y de Misahuallí; Jardin Aleman, Misahualli; Pununo; Road to Shalcana 2; Road to Shalcana	33	25	-1.078	-77.624	414
bar	West	Colombia	Low	Santa Barbara	0	1	3.830	-76.786	282
cau	West	Colombia	Low	La Elsa - Rio Digua	0	7	3.580	-76.862	346
all	West	Ecuador	Low	Alluriquin	0	2	-0.320	-79.337	300
gua	West	Ecuador	Low	Guayllabamba	0	12	0.190	-78.902	532
tor	West	Ecuador	Low	Mashpi town (117); El Tortugo, P28; El Tortugo, P26; El Tortugo, P25; El Tortugo, P24; Pachijal to Tortugo 6	32	11	0.204	-78.943	480
amz	East	Colombia	Low distant	Puerto Nari_o-Quebrada aguas rojas; Puerto Nari–o- Quebrada aguas rojas	6	1	-3.770	-70.340	101
let	East	Colombia	Low distant	Leticia - Vereda San Jose; Leticia - Km 9.3; Leticia - Reserva Cerca viva km12; Leticia - Tio Tacana 2km	16	7	-4.147	-69.960	50
ana	East	Ecuador	Low distant	Anangu Boca del Rio ECD OR; Napo Wildlife Center community Anangu	14	5	-0.504	-76.388	228
yas	East	Ecuador	Low distant	Yasuni Research Station,	14	0	-0.674	-76.397	230
pot	West	Colombia	Low distant	Potes 2 ; Playa Flores 3; Playa Flores; Cocalito; Cocalito 2	0	13	6.375	-77.387	30
ama	West	Colombia	Low distant	Amargal Station; Amargal 1	4	5	5.572	-77.502	72
ped	West	Colombia	Low distant	San Pedro 1	4	0	3.837	-77.257	4
lad	West	Colombia	Low distant	Ladrilleros; La Barra	16	4	3.958	-77.373	34
pue	West	Ecuador	Low distant	Puerto Quito, La Isla	16	0	0.110	-79.230	130
ton	West	Ecuador	Low distant	Tonsupa 1	22	0	0.854	-79.804	77

Supplementary Table 2. Mean genome-wide Tajima's and nucleotide diversity (pi) per population, calculated in 5kb windows with 1kb steps.

Species	Pop.	pi	Tajima's D	Cline	Altitude
H. erato	que	0.024	-0.444	Col. West	High
	min	0.022	-0.504	Ec. West	High
	pac	0.023	-0.670	Ec. West	High
	tor	0.023	-0.834	Ec. West	Low
	lad	0.023	-0.775	Col. West	Low distant
	pue	0.023	-0.708	Ec. West	Low distant
	ton	0.023	-0.734	Ec. West	Low distant
	cam	0.032	-0.751	Col. East	High
	moc	0.031	-0.580	Col. East	High
	nar	0.032	-1.135	Ec. East	High
	rev	0.032	-1.015	Ec. East	High
	esc	0.033	-1.317	Col. East	Low
	guz	0.033	-1.146	Col. East	Low
	jat	0.033	-1.392	Ec. East	Low
	amz	0.032	-0.851	Col. East	Low distant
	let	0.034	-1.264	Col. East	Low distant
	ana	0.033	-1.267	Ec. East	Low distant
	yas	0.034	-1.210	Ec. East	Low distant
H. melpomene	pac	0.020	-0.065	Ec. West	High
	bra	0.019	0.301	Col. West	High
	gua	0.020	0.067	Ec. West	Low
	tor	0.019	-0.068	Ec. West	Low
	cau	0.020	0.170	Col. West	Low
	ama	0.020	0.068	Col. West	Low distant
	pot	0.021	-0.221	Col. West	Low distant
	bae	0.019	-0.260	Ec. East	High
	nar	0.021	-0.566	Ec. East	High
	sum	0.020	-0.278	Ec. East	High
	cam	0.021	-0.610	Col. East	High
	flo	0.021	-0.391	Col. East	High
	jat	0.021	-1.061	Ec. East	Low
	guz	0.020	-0.479	Col. East	Low
	ana	0.021	-0.522	Ec. East	Low distant
	let	0.021	-0.561	Col. East	Low distant

Supplementary Table 3. Local PCA summaries across different types of SHDRs. Linear models were built with local PCA PC1 as a response variable and individual altitude and global (genome-wide) neutral PCA PC1 as explanatory variables, i.e. accounting for genome-wide population structure. Partial R^2 for altitude was calculated with the package *relaimpo* [19].

SHDR type	No. SHDR (no. that are also allopatric SHDRs)	% SHDR with altitude as a significant predictor of local PCA PC1	% allopatric SHDR with significant correlation between PC1 and altitude	Mean % of variation explained by local PC1 (global PC1, for comparison)	Mean altitude partial R^2 for models where altitude was a significant predictor of local PCA PC1 (altitude partial R^2 in global PC1 model, for comparison)
H. erato West	133 (38)	25%	16%	58% (21%)	0.05 (n.s.)
H. erato East	75 (38)	48%	32%	55% (8.8%)	0.16 (0.11)
H. melpomene West	104 (11)	74%	55%	56% (36%)	0.10 (n.s.)
H. melpomene East	58 (11)	66%	73%	66% (11%)	0.15 (n.s.)

Supplementary Figures

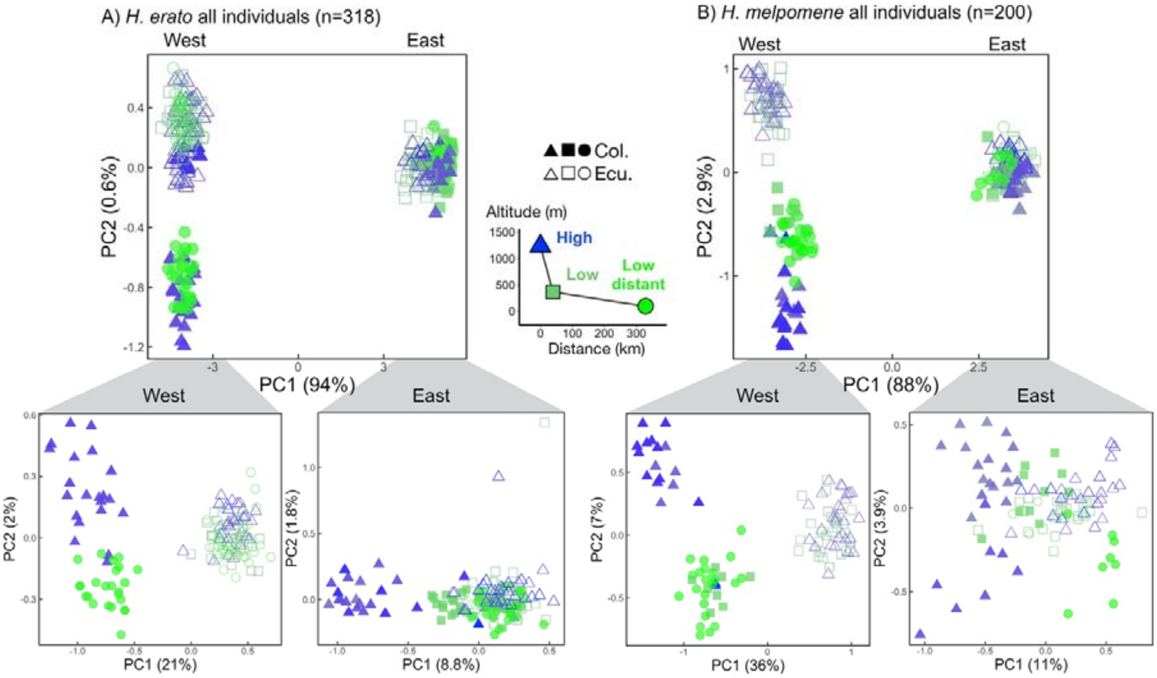

Supplementary Figure 1. Population structure across levels of geographic isolation. Population structure between all individuals of *H. erato* and *H. melpomene* (top panel) and divided into sides of the Andes (bottom panel). Genetic variation along the first two eigenvectors of a principal component analysis (PCA) and the percentage of variance explained by the principal components is shown in brackets. Note that in the West, Ecuador and Colombia have different subspecies/colour patterns (Fig. 1D), and, additionally, the highlands of Colombia East have (filled blue triangles) different subspecies compared to the lowlands.

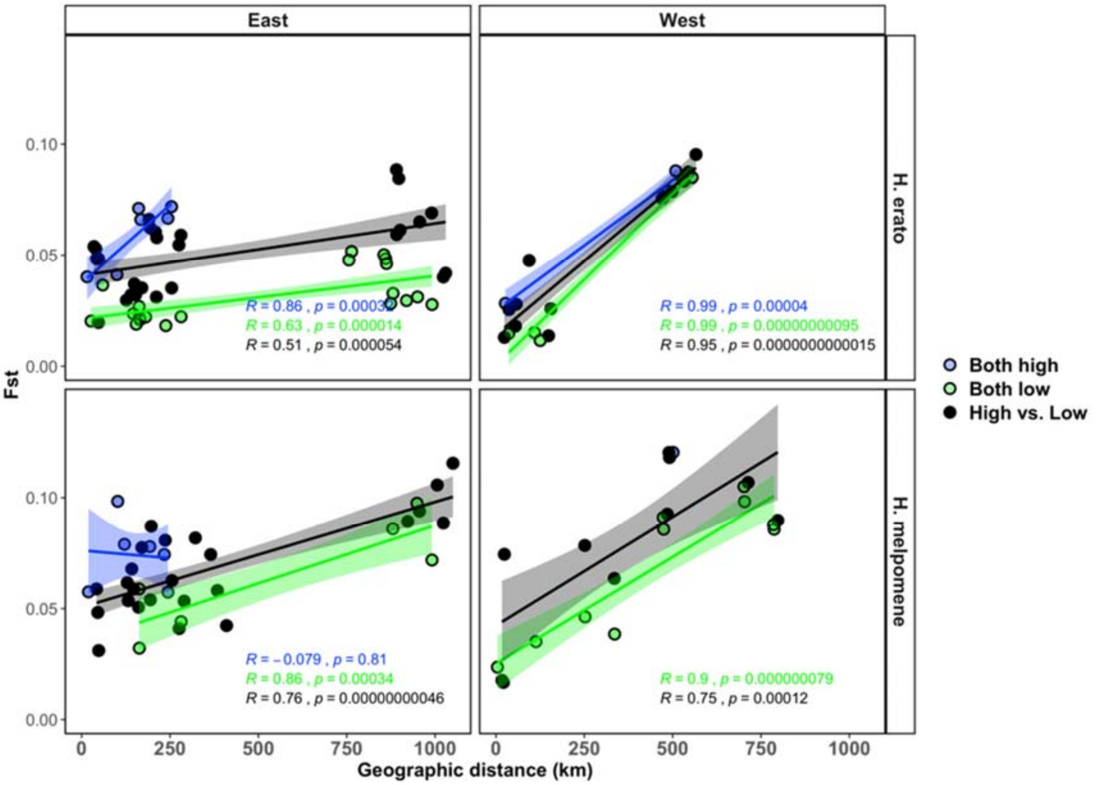

Supplementary Figure 2. Genetic vs geographic distance. Pairwise population genetic differentiation (Fst) between all population pairs per side of the Andes and per species. Population pairs can be both at high altitude (blue), both at low altitude (green), and high vs. low altitude (black). Geographic distance incorporates topography and least cost paths to circumvent elevations at which these species are not found. Pearson correlation and p-values shown. Solid lines represent best fit of linear models per group, with the shaded area showing confidence bands at 1 standard error. Pearson correlation coefficients (R) and their significance are shown as text.

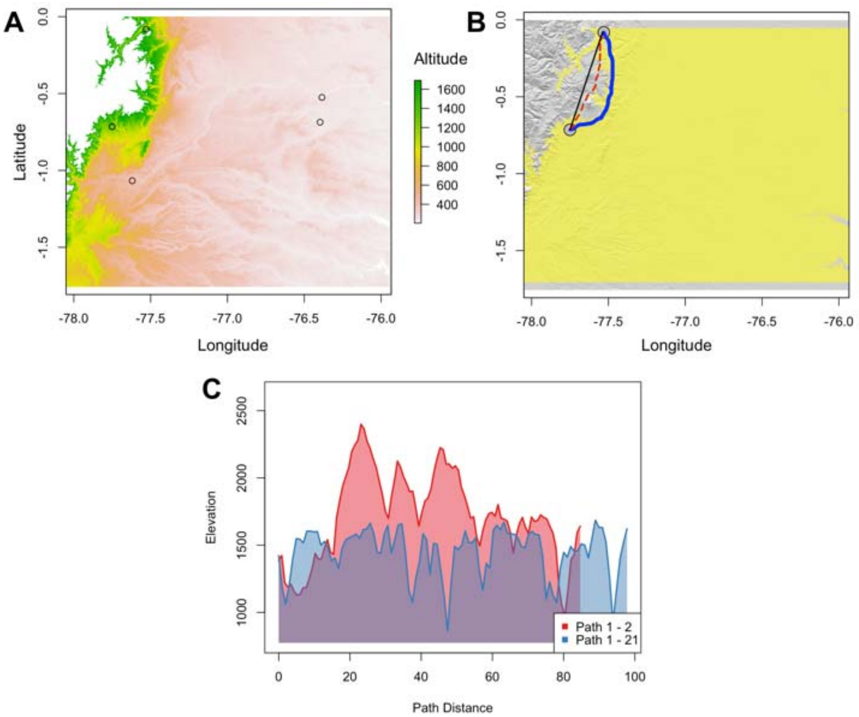

Supplementary Figure 3. Least cost path distance example. A) Elevation raster obtained with the package elevatr [20] for highland and lowland Ecuador eastern populations (circles) with altitudes covering the elevational ranges of *H. erato* (Map data ©2019 Google obtained via RgoogleMaps package[8]). B) Geographic distance as a straight line between populations (black line), topographic distance without accounting for elevational ranges (red dotted line), and least cost path topographic distance accounting for species elevational rages (blue solid line). The Sumaco Volcano (Napo, Ecuador) is located between these points. C) Comparison of topographic distance (red) and least cost path topographic distance (blue).

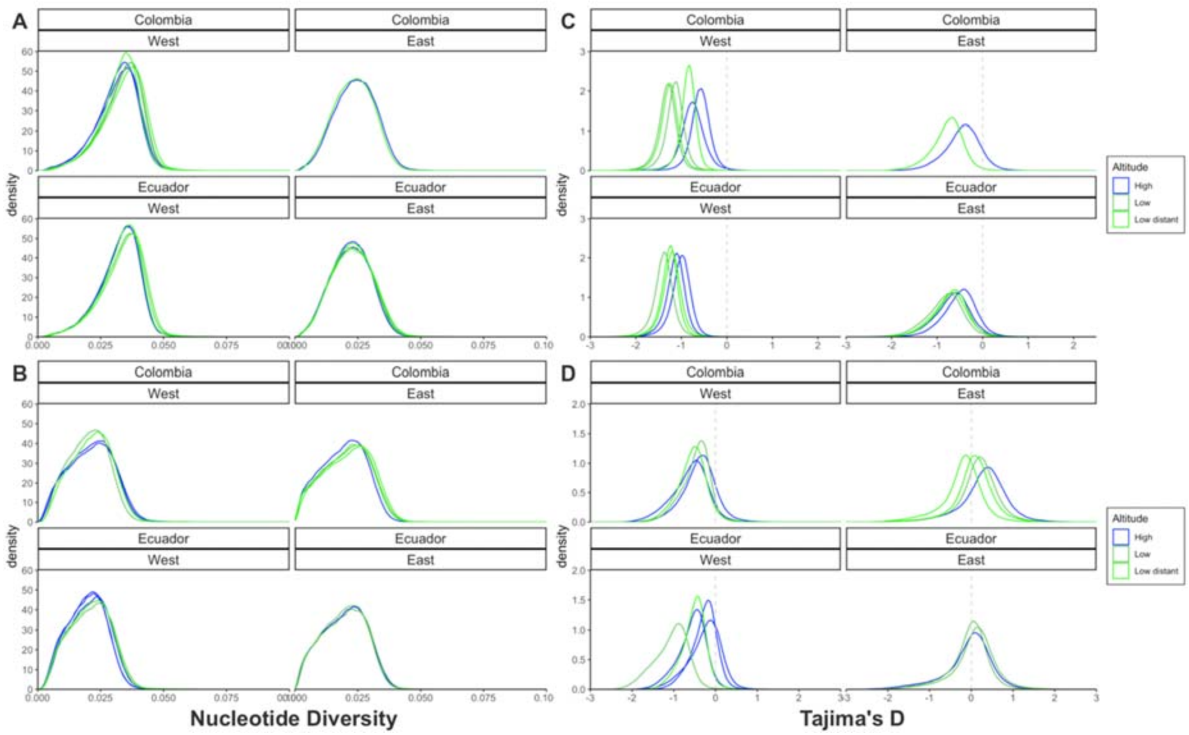

Supplementary Figure 4. Overall patterns of diversity and neutrality. Nucleotide diversity (π, A, B) and Tajima’s D (C, D) for *H. erato* (A, C) and melpomene (B, D) calculated per population (Supplementary Table 1), and coloured by population area (highland, lowland, lowland distant) and by side of the Andes / species.

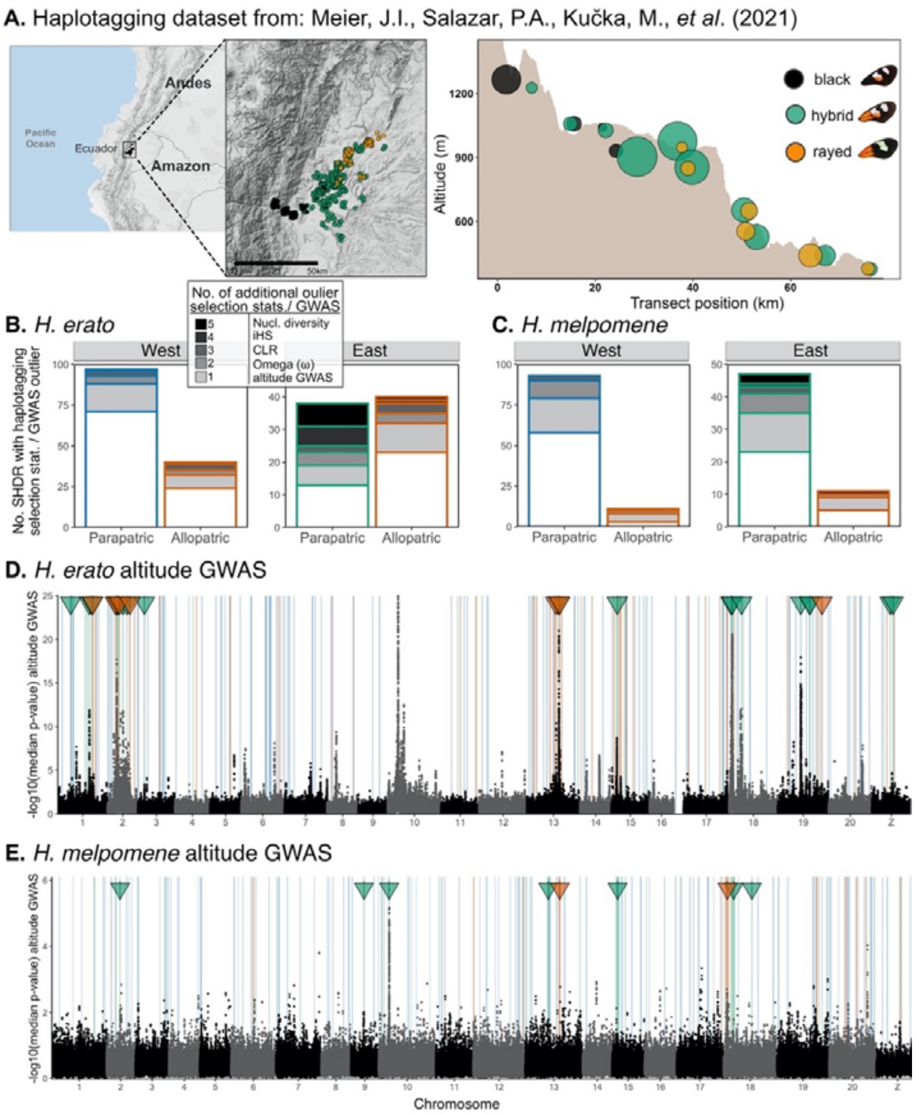

Supplementary Figure 5. Validation of SHDRs with independent published haplotagging sequencing dataset from an altitudinal cline on the Eastern Ecuadorian Andes. A) Each point represents an individual butterfly collected in the wild and sequenced in Meier et al., 2021). Map data ©2019 Google obtained via RgoogleMaps package[8]. The panel on the right shows the distribution of individuals sequenced across elevations. *H. erato* and H. melpomene co-occur and have three main colour pattern morphs along this cline: two distinct colour pattern morphs (*H. e notabilis* and *H. m. plesseni*, referred to as "black", and *H. e lativitta* and *H. m. malletti*, referred to as "rayed") and within-species hybrids displaying admixed phenotypes (green circles), the most common hybrid phenotype is depicted. Adapted from Montejo-Kovacevich *et al.* 2021. B-C) Number of altitude SHDR which overlap with selection statistic outliers or genome-wide association regions obtained from the analysis of the published haplotagging dataset. Darker categories indicate more selection statistics overlap (white=0 additional haplotagging selection statistics, black= 5 outlier statistics overlap). Selection statistics included: integrated haplotype score (iHS), nucleotide diversity (Π) across elevations ($\Delta\Pi = \Pi_{high} - \Pi_{low}$), omega (ω), composite likelihood ratio to detect selective sweeps using sweeD, and altitude GWAS regions (Meier *et al*. 2021). D-E) Altitude genome-wide association study of haplotagging dataset with parapatric eastern (green), parapatric western (blue), or allopatric (red) SHDRs highlighted as vertical lines. The genome-wide 90th quantile threshold was obtained with 200 permutations of the phenotype, resulting in thresholds of -log10(median p-value)=2.9 in *H. erato*, and -log10(median p-value)=1.8 in *H. melpomene*. Arrows indicate SHDRs that overlap with outlier altitude-associated regions.

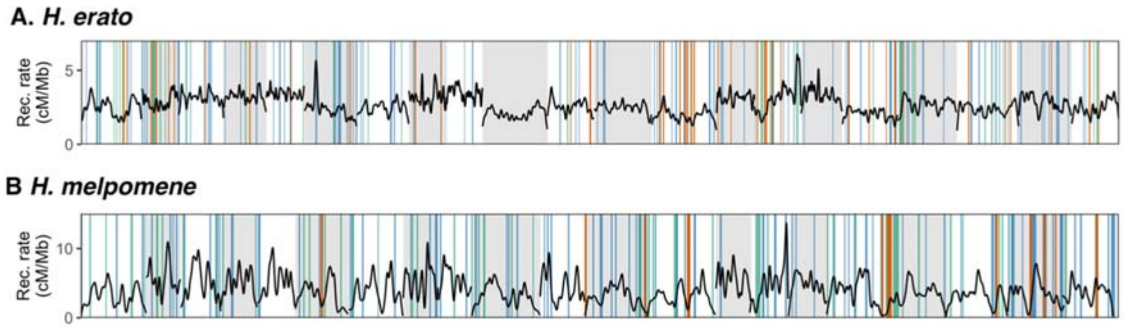

Supplementary Figure 6. Recombination rates (cM/Mb) across the genome for *H. erato* (A) and *H. melpomene* (B). SHDR highlighted as vertical lines in blue/green if shared across parapatric transects of the same species, i.e. within sides of the Andes (SHDR: blue=within West, green=within East) and in red those additionally shared across parapatric transects within species, i.e. shared across all four transects (also SHDR).

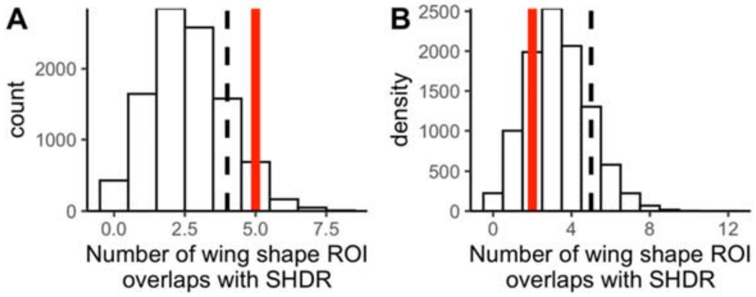

Supplementary Figure 7. Wing shape outlier regions overlapping with SHDR. Number of wing shape regions of interest (ROI) identified in a previous study that overlap with H. erato (A) and H. melpomene (B) SHDR. Observed values are shown as red vertical lines. Bars represent 10,000 simulations, where the same number of ROI and of the same size were placed in random non-overlapping positions across the genome. Black dashed vertical line represents 90th quantile of simulations.

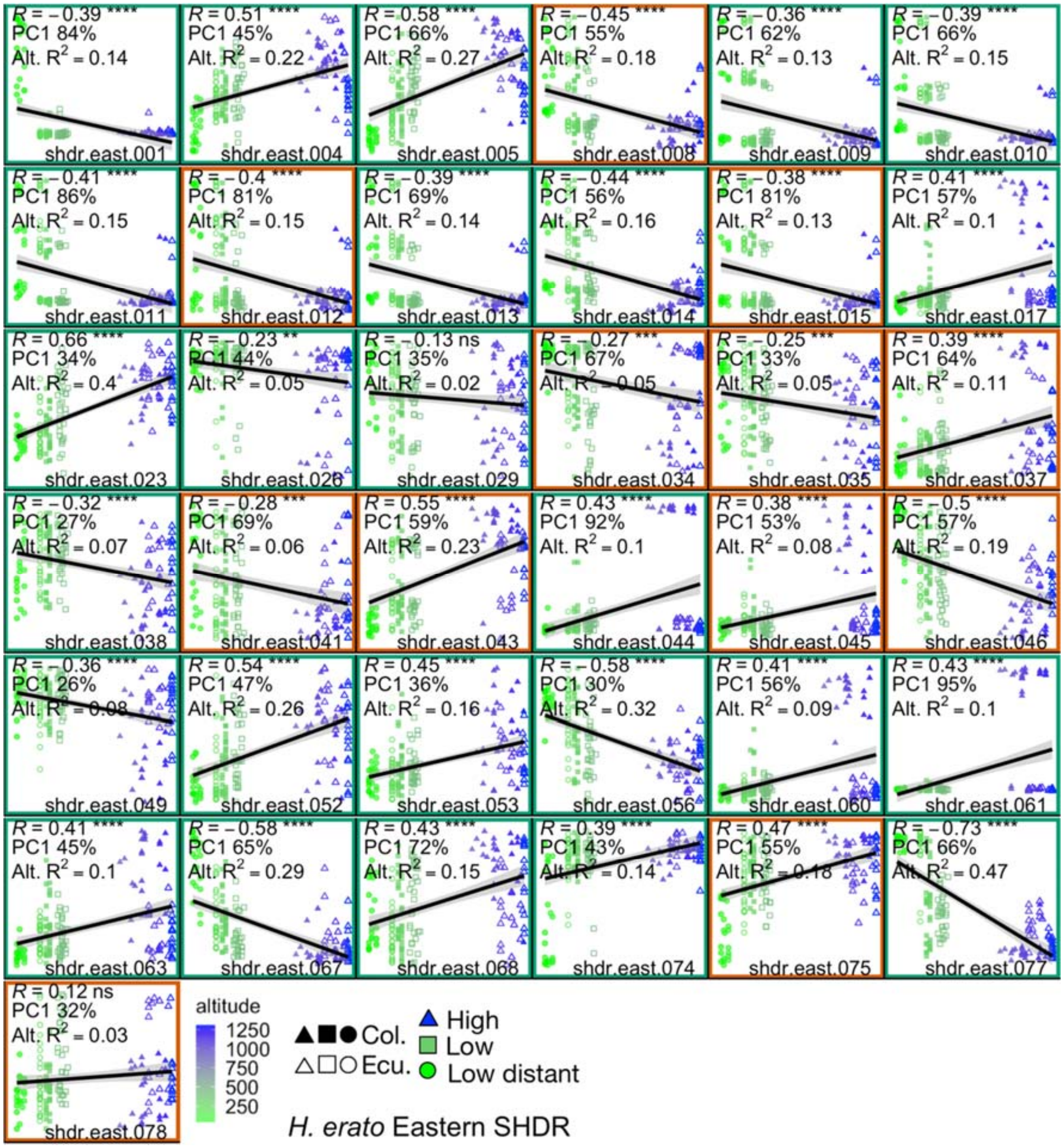

Supplementary Figure 8. Local PCA PC1 axes (y-axes) of SHDR correlate with altitude (x-axes). *H. erato* eastern Shared High Differentiation Regions (SHDR) for which altitude was a significant predictor (P<0.05) of local PCA PC1, while accounting for neutral genome wide PCA PC1, i.e. population structure. SHDRs local PCAs included genomic windows found to be $zPBS_{high}$ outliers (>4stdv from the mean) in either Colombian or Ecuadorian clines. Each point is an individual; when solid from Colombia and empty from Ecuador. SHDRs additionally shared with the western clines (allopatric SHDRs) are highlighted with red boxes. Altitude is indicated by colour and symbols. Green plot borders indicate SHDRs shared across replicate transects within the East of the Andes and in red those additionally shared across allopatric transects, i.e. shared across all four transects. Each SHDR plot shows: percentage of the total variation explained by PC1 (y-axes), pearson correlation coefficients (R) of the line shown (p-values for the correlations as stars ****P<0.0001, ***P<0.001, **P<0.01, *P<0.05), and the partial variation (R^2) explain by altitude in the model that accounts for accounting for neutral genome-wide PCA PC1. Note that *H. erato* color patter loci overlap with shdr.east.039, shdr.east.044, shdr.east.061, and the chromosome 2 inversion presented in the main text overlaps with shdr.east.008-015.

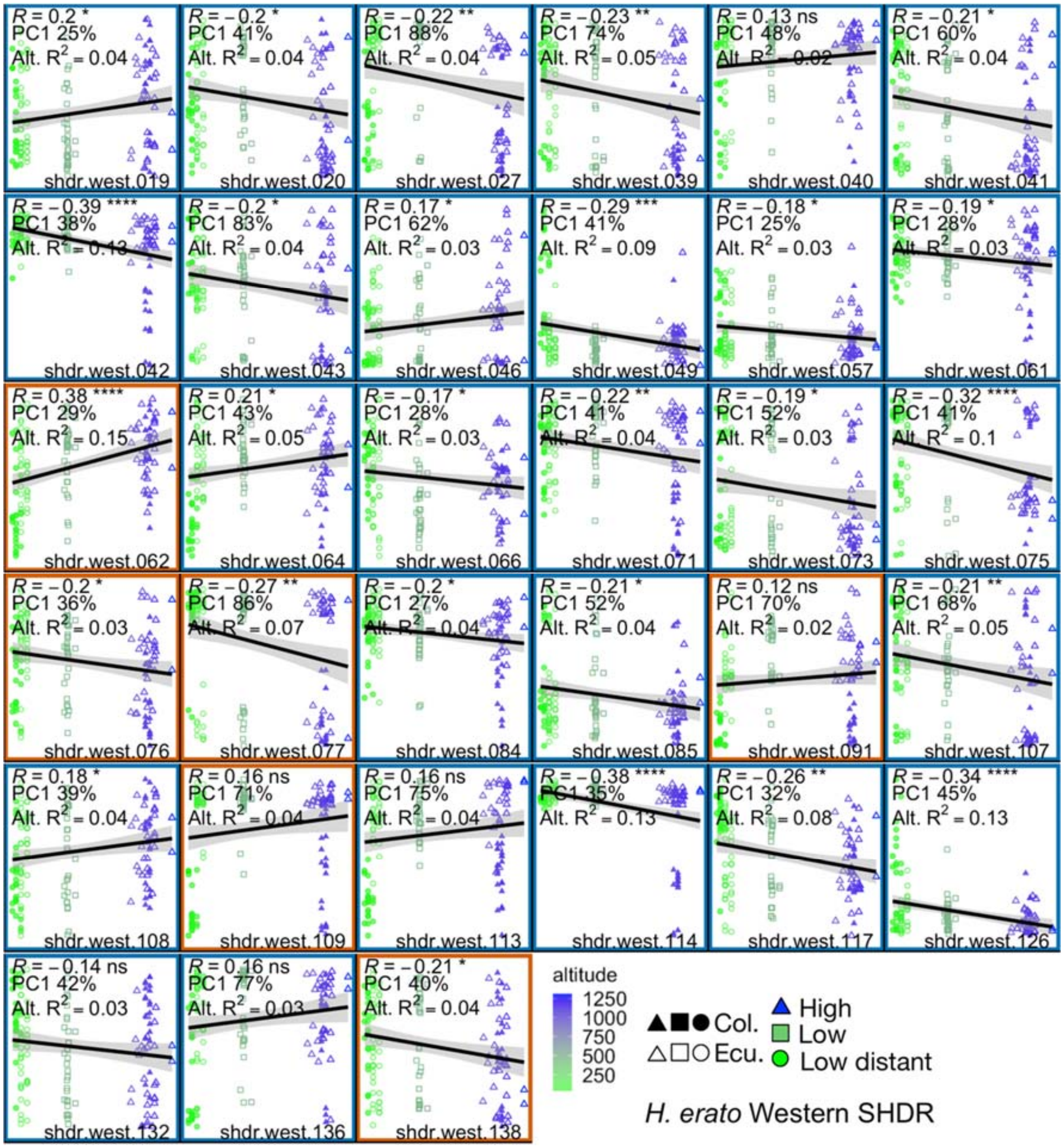

Supplementary Figure 9. Local PCA PC1 axes (y-axes) of SHDR correlate with altitude (x-axes). *H. erato* western Shared High Differentiation Regions (SHDR) for which altitude was a significant predictor (P<0.05) of local PCA PC1, while accounting for neutral genome wide PCA PC1, i.e. population structure. SHDRs local PCAs included genomic windows found to be $zPBS_{high}$/zFst outliers (>4stdv from the mean) in either Colombian or Ecuadorian clines. Each point is an individual; when solid from Colombia and empty from Ecuador. Altitude is indicated by colour and symbols. Blue plot borders indicate SHDRs shared across replicate transects within the West of the Andes and in red those additionally shared across allopatric transects, i.e. shared across all four transects. Each SHDR plot shows: percentage of the total variation explained by PC1 (y-axes), pearson correlation coefficients (R) of the line shown (p-values for the correlations as stars ****P<0.0001, ***P<0.001, **P<0.01, *P<0.05), and the partial variation (R^2) explain by altitude in the model that accounts for accounting for neutral genome-wide PCA PC1.

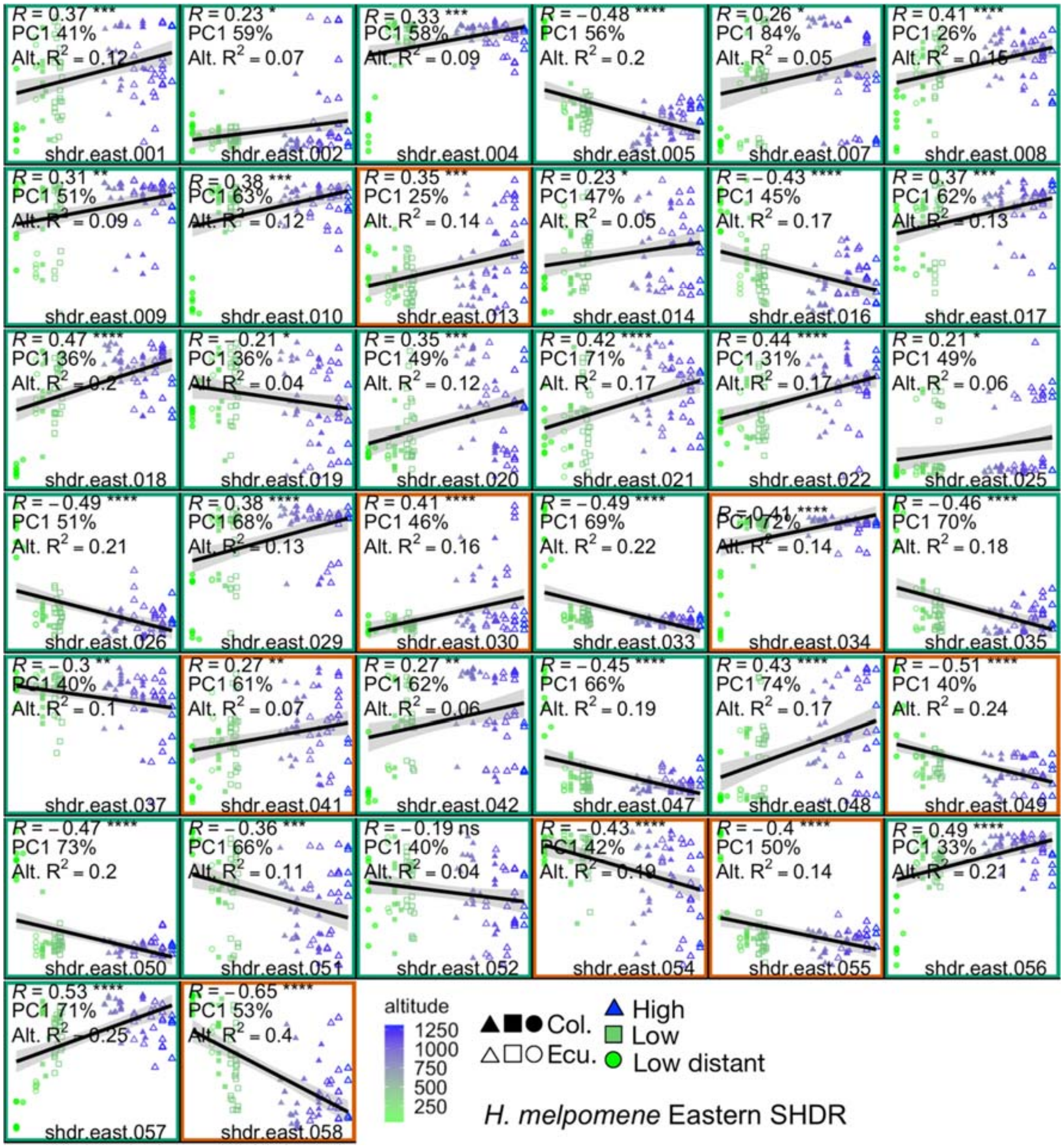

Supplementary Figure 10. Local PCA PC1 axes (y-axes) of SHDR correlate with altitude (x-axes). *H. melpomene* eastern Shared High Differentiation Regions (SHDR) for which altitude was a significant predictor (P<0.05) of local PCA PC1, while accounting for neutral genome wide PCA PC1, i.e. population structure. SHDRs local PCAs included genomic windows found to be $zPBS_{high}$/zFst outliers (>4stdv from the mean) in either Colombian or Ecuadorian clines. Each point is an individual; when solid from Colombia and empty from Ecuador. Altitude is indicated by colour and symbols. Green plot borders indicate SHDRs shared across replicate transects within the East of the Andes and in red those additionally shared across allopatric transects, i.e. shared across all four transects. Each SHDR plot shows: percentage of the total variation explained by PC1 (y-axes), pearson correlation coefficients (R) of the line shown (p-values for the correlations as stars ****P<0.0001, ***P<0.001, **P<0.01, *P<0.05), and the partial variation (R^2) explain by altitude in the model that accounts for accounting for neutral genome-wide PCA PC1. Note that melpomene color patter loci overlap with shdr.east.024, shdr.east.036, shdr.east.042, shdr.east.043.

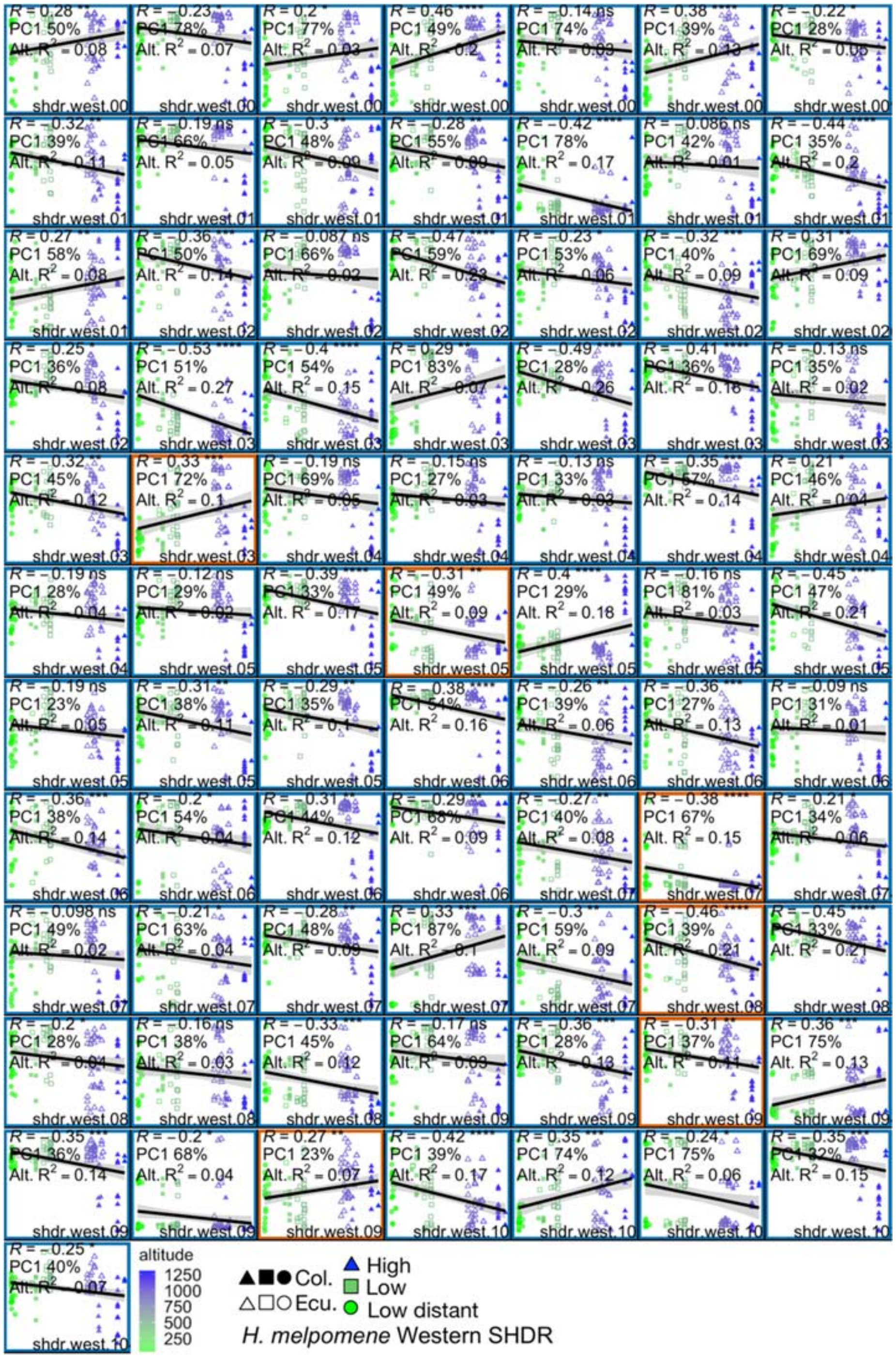

Supplementary Figure 11. Local PCA PC1 axes (y-axes) of SHDR correlate with altitude (x-axes). *H. melpomene* western Shared High Differentiation Regions (SHDR) for which altitude was a significant predictor (P<0.05) of local PCA PC1, while accounting for neutral genome wide PCA PC1, i.e. population structure. SHDRs local PCAs included genomic windows found to be $zPBS_{high}$/zFst outliers (>4stdv from the mean) in either Colombian or Ecuadorian clines. Each point is an individual; when solid from Colombia and empty from Ecuador. Altitude is indicated by colour and symbols. Blue plot borders indicate SHDRs shared across replicate transects within the West of the Andes and in red those additionally shared across allopatric transects, i.e. shared across all four transects. Each SHDR plot shows: percentage of the total variation explained by PC1 (y-axes), pearson correlation coefficients (R) of the line shown (p-values for the correlations as stars ****P<0.0001, ***P<0.001, **P<0.01, *P<0.05), and the partial variation (R^2) explain by altitude in the model that accounts for accounting for neutral genome-wide PCA PC1.

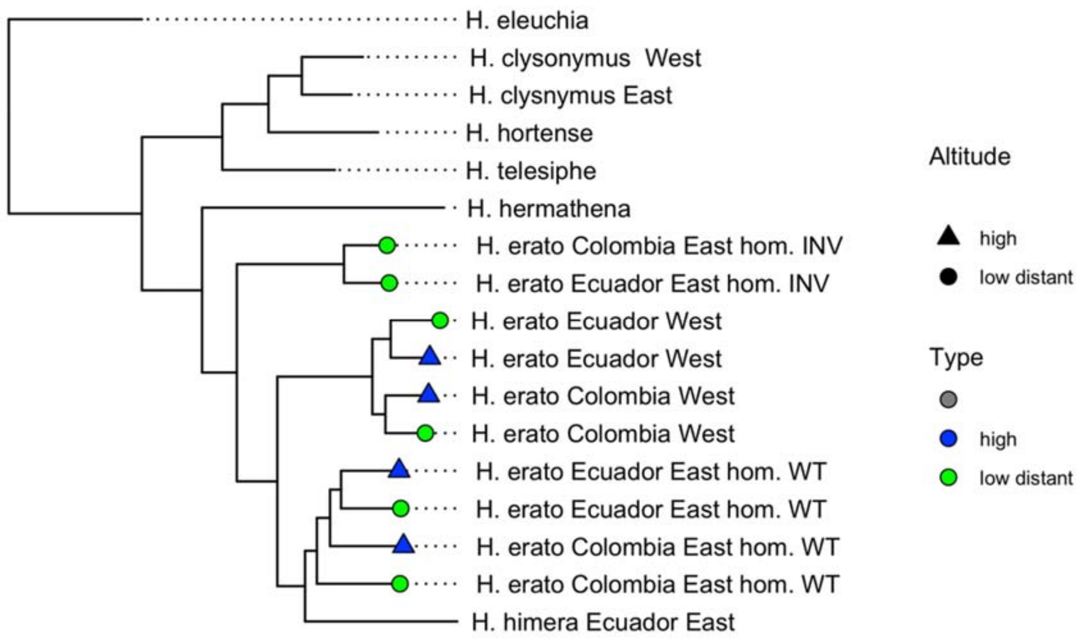

Supplementary Figure 12. Local neighbour joining tree of large putative inversion in chromosome 2 of H. erato H. erato0204:1293500 - H. erato0215:2099500. Inversion haplotypes of eastern *H. erato* were inferred from local PCA presented in Fig 4B, INV for inverted and WT for wild-type.

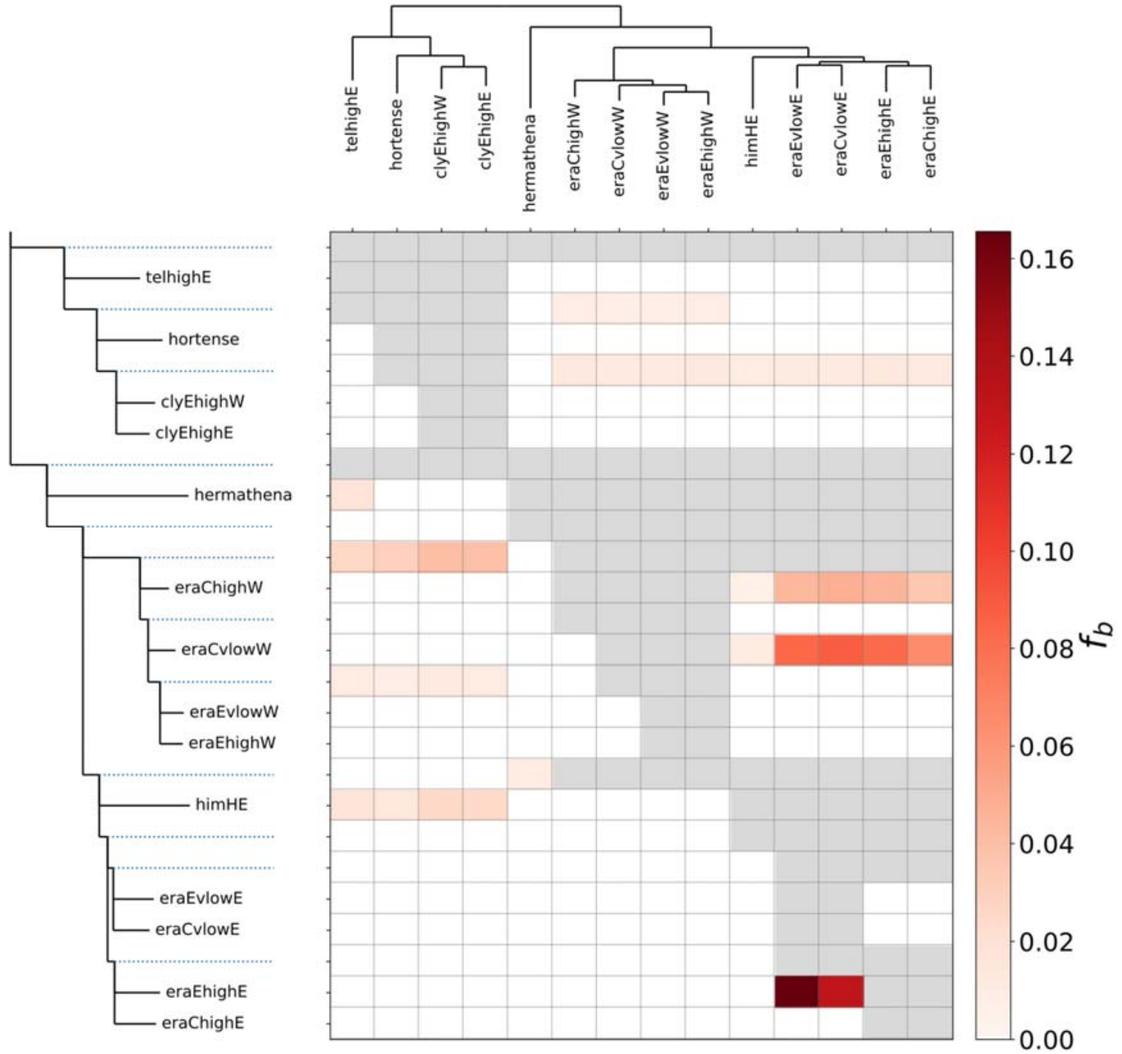

Supplementary Figure 13. Fbranch statistics obtained with the package *DSuite* [22] for *H. erato* relatives. The 'true' tree, i.e. the most frequent tree topology genome-wide, is shown along x-axis, and is considered P3 when obtaining f-branch statistics. This tree is then expanded along the y-axis, so that each branch, including internal ones, has a row in the matrix with the inferred f-branch statistic. The values in the matrix thus refer to excess allele sharing between the branch b identified on the expanded tree on the y-axis (relative to its sister branch) and the species P3 identified on the x-axis. Non-significant f4-ratio values are set to zero prior to obtaining f-branch statistics, thus all f-branch values>0 had significant associated D statistics (p<0.001), indicating excess allele sharing for that trio.The first three letters of the tip labels correspond to species names (cly: H. clysonymus, era: *H. erato*, him: H. himera), the fourth letter, if present, indicates whether the sample was collected in Colombia (C) or Ecuador (E), then whether it was collected in the higlands (high) or lowland distant (vlow) populations, and finally the last letter indicates side of the Andes (E: east, and W: West). Outgroups are represented by the species name only.

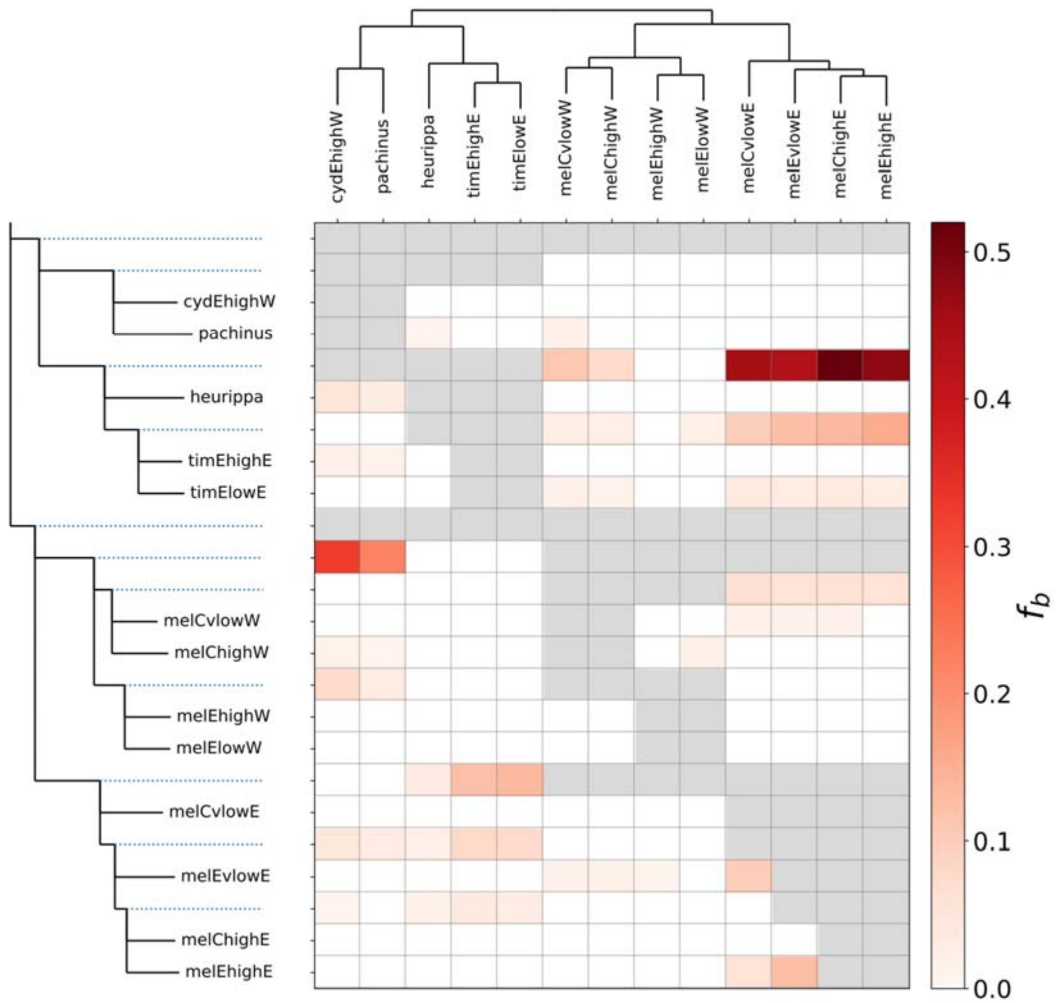

Supplementary Figure 14. Fbranch statistics obtained with the package DSuite [22] for melpomene relatives. See explanation of axes in Supplementary Figure 7. The tree was constrained to fit the species phylogeny from Kozak *et al*., 2015, to ensure that the high levels of gene flow between H. timareta and H. melpomeneThe first three letters of the tip labels correspond to species names (cyd: *H. cydno*, mel: melpomene, tim: *H. timareta*), the fourth letter, if present, indicates whether the sample was collected in Colombia (C) or Ecuador (E), then whether it was collected in the higlands (high) or lowland distant (vlow) populations, and finally the last letter indicates side of the Andes (E: east, and W: West). Outgroups are represented by the species name only.

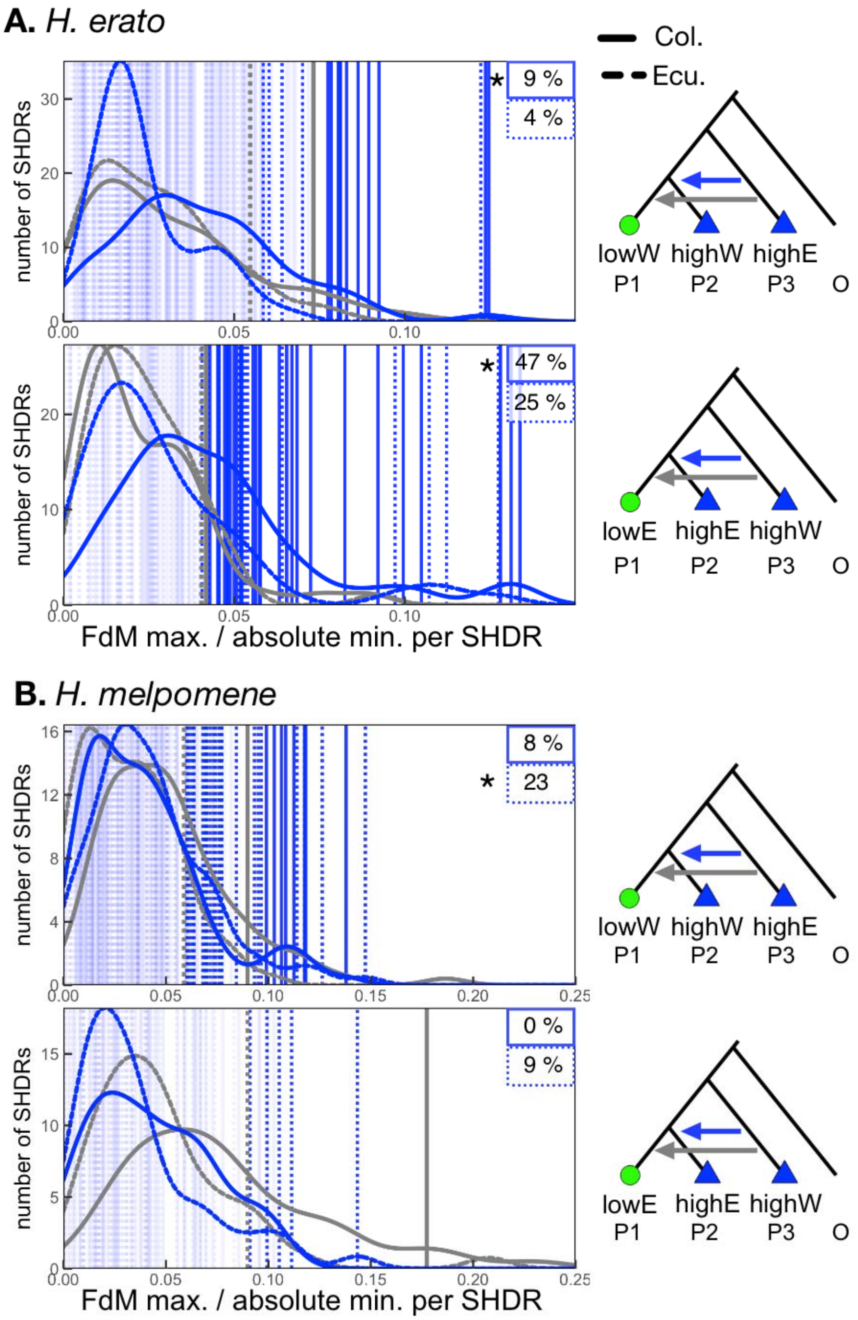

Supplementary Figure 15. Putative shared ancestral variation within high altitude shared adaptive regions (SHDRs). Excess allele sharing between highland populations on opposite sides of the Andes, for *H. erato* (A) or *H. melpomene* (B) populations (FdM>0; blue triangles in trees) or with lowland populations (FdM<0, absolute values represented with grey lines; green circles in trees). Lowland populations correspond to lowland distant populations, except in *H. melpomene* Ecuador where only lowland populations near highlands were available. Outlier maximum FdM values per SHDR are highlighted with darker vertical lines (>90th of the neutral distribution of minimum FdM values, in grey), dashed if detected in the Colombian cline or solid if in the Ecuadorian cline. Percentage of SHDRs with outlier maximum FdM values per cline and comparison is shown on the top right corners of each plot. Significant two-sample Kolmogorov-Smirnov tests comparing overall SHDR FdM_{max} and absolute FdM_{min} distributions are indicated by stars (P-values from top to bottom: 0.00046, $1.6e^{-5}$, 0.00031;).

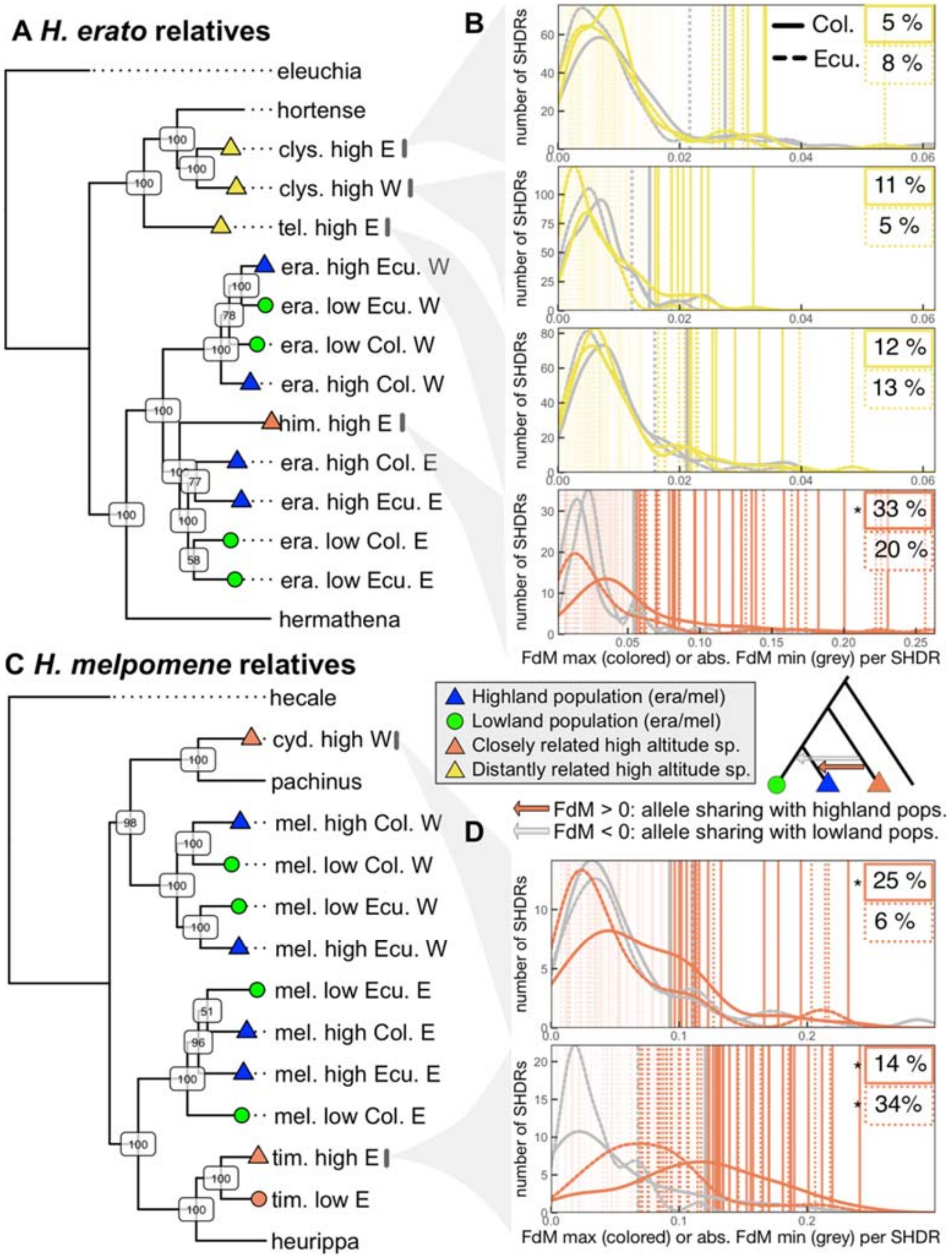

Supplementary Figure 16. Putative shared ancestral variation within high altitude shared adaptive regions (SHDRs) with related highland species. Excess allele sharing with highland *H. erato* (B) or *H. melpomene* (D) populations (FdM>0) or with lowland populations (FdM<0, absolute values represented with grey lines). Outlier maximum FdM values per SHDR are highlighted with darker vertical lines (>90th of the neutral distribution of minimum FdM values, in grey), solid if detected in the Colombian cline or dashed if in the Ecuadorian cline. Percentage of SHDRs with outlier maximum FdM values per cline and comparison is shown on the top right corners of each plot. Donor species (P3) of each plot in B and D is indicated with an arrow to the trees in A and C. Only parapatric allele sharing comparisons are shown, i.e. those where the recipient *H. erato* or *H. melpomene* populations were on the same side of the Andes as the high altitude species donor. Significant two-sample Kolmogorov-Smirnov tests comparing overall SHDR FdM_{max} and absolute FdM_{min} distributions are indicated by stars (P-values from top to bottom: $2.0e^{-7}$, $2.6e^{-8}$, 0.007, 2.0^{-10}).

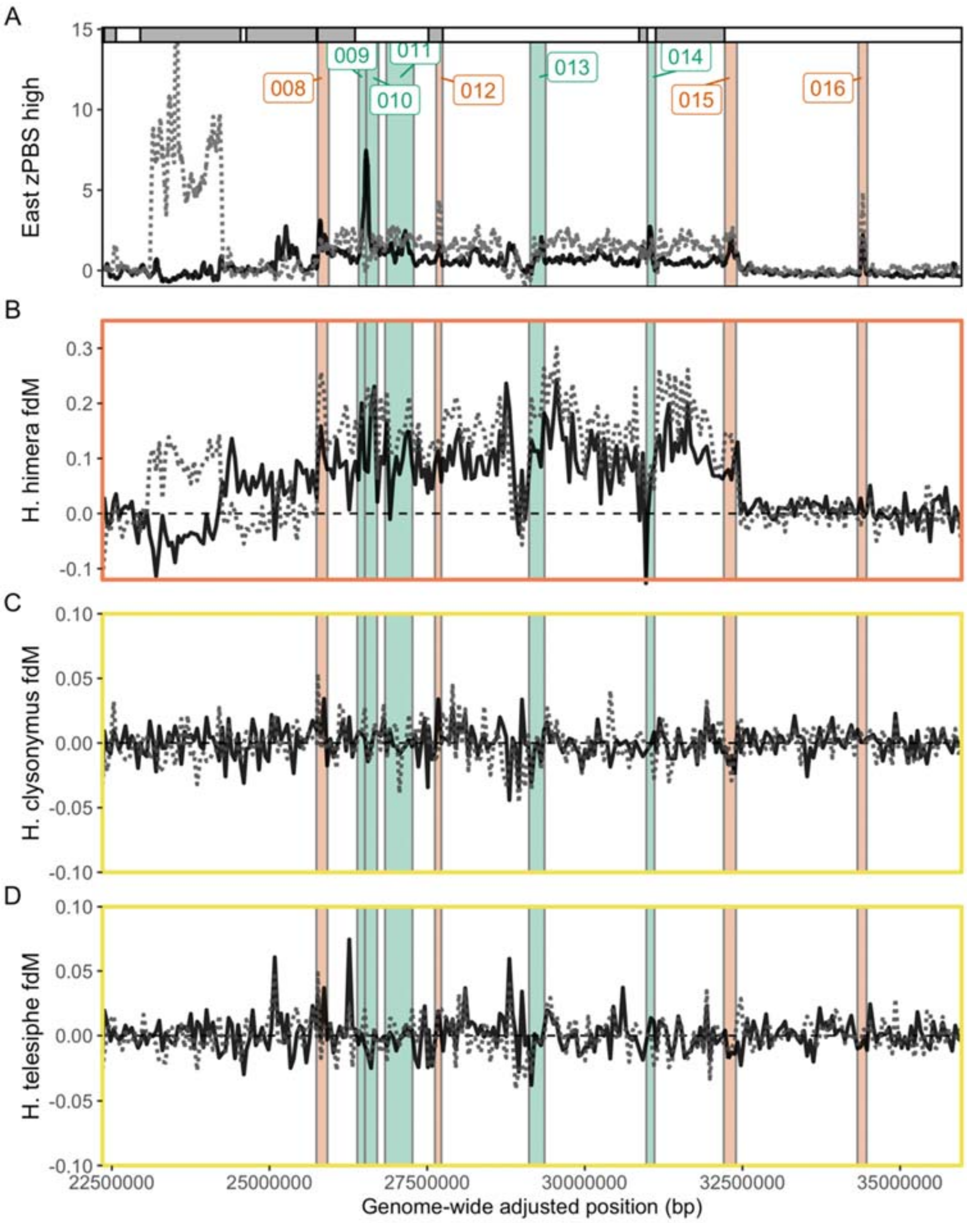

Supplementary Figure 17. SHDRs across *H. erato* chromosome 2. A) zPBS along chromosome 2, scaffolds indicated as grey/white segments and *H. erato* Colombian cline is a dashed black line whereas Ecuador cline is grey and dashed. First elevated zPBS region, exclusive to the Ecuadorian cline, is a confirmed inversion identified in Meier et al. 2021. Second elevated region and putative inversion is presented in the main text. Excess allele sharing between highland relatives (P3: B *H. himera*, C *H. clysonymus*, D *H. telesiphe*) and highland *H. erato* eastern populations (FdM>0) or with lowland populations (FdM<0) in *H. erato* chromosome 2. Solid black lines represent comparisons that include the *H. erato* Colombian cline or dashed if in the Ecuadorian cline. Shared High Differentitation Regions (SHDRs) are shown as numbered vertical segments, colored by whether they represent parapatric SHDRs within Eastern clines (green) or allopatric shared across all clines (red, SHDRs shown in Fig. 2). The orange box around A denotes that *H. himera* is a closely-related species of *H. erato*, whereas in yellow are distantly related species (see Supplementary Figure 15).

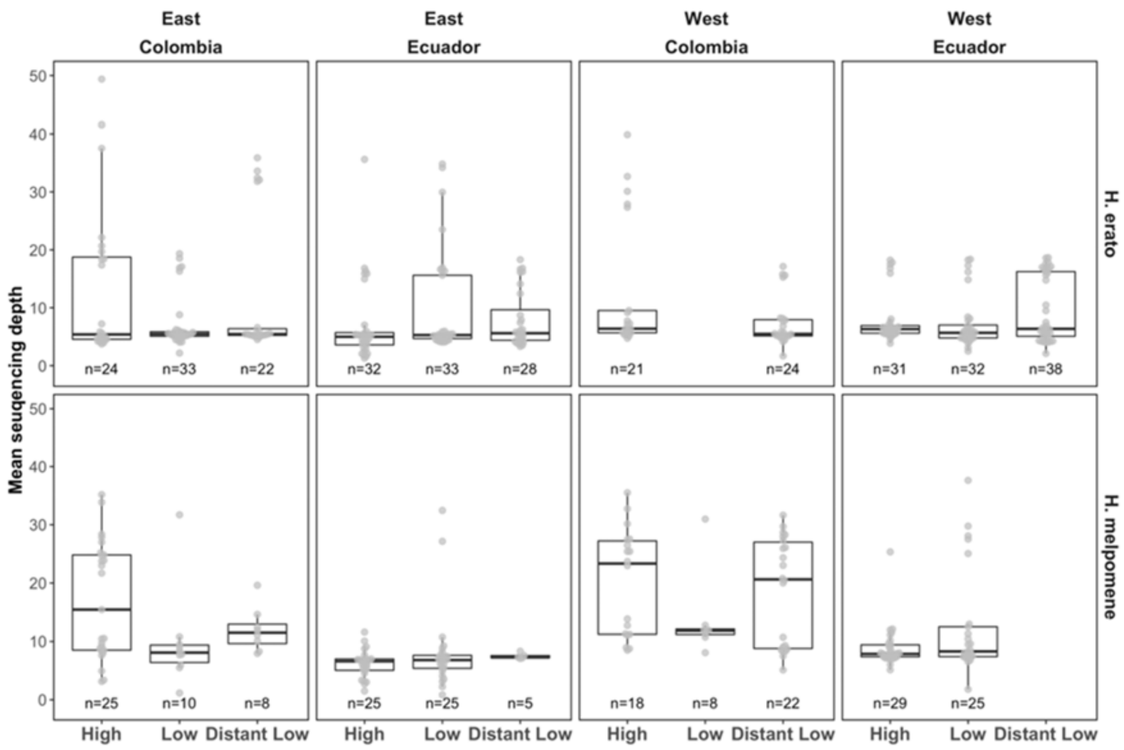

Supplementary Figure 18. **Mean genome-wide sequencing depth per individual across clines, countries, sides of the Andes and species**. Number of individuals (biological replicates) indicated under each box plot (n). Genome-wide mean depth per individual calculated with samtools[23]. The bottom and top of the boxes represent the first and third quartiles, respectively, the bold line represents the median, the grey points represent the raw data, and the vertical line delimits maximum and minimum non-outlier observations.

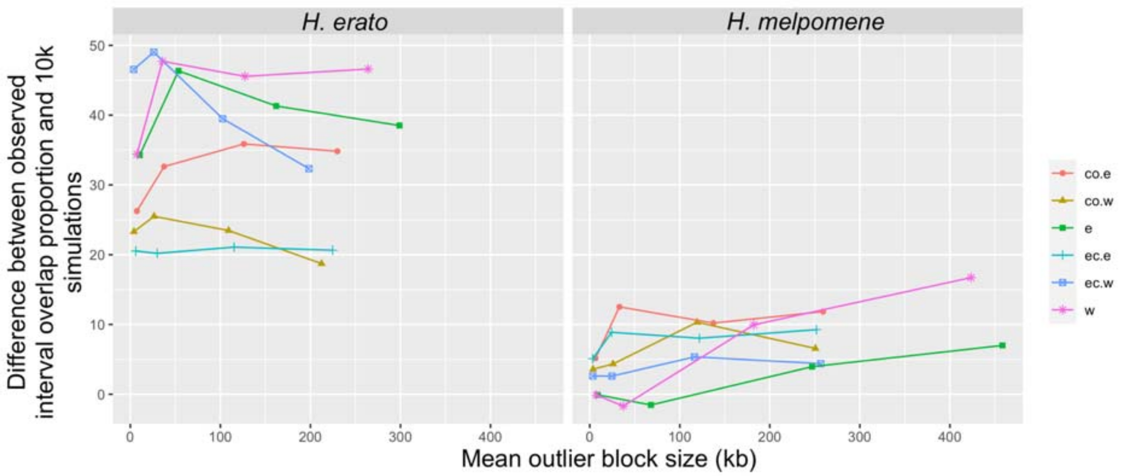

Supplementary Figure 19. Effect of increasing outlier window buffer size on the difference between the observed HDR overlap proportion between clines and the simulated null distribution of such proportions. Each point (from left to right) represents buffers (±) of size: 0kb, 10kb, 50kb, 100kb, which results in larger HDRs (x-axis). Importantly, the difference between the observed and the simulated proportion, i.e. the significance of parallelism, only increases in *H. melpomene*, when using 100kb buffers. Each colour is a different cline population compared against the other cline on the same side of the Andes (when the code is “country.side”; co=Colombia, ec=Ecuador, e=East, w=West) or compared against the clines on the other side of the Andes (when the code is “side”).

References

1. Jiggins, C. D. *The Ecology and Evolution of Heliconius Butterflies*. (Oxford University Press, 2016).
2. Kozak, K. M. *et al.* Multilocus species trees show the recent adaptive radiation of the mimetic heliconius butterflies. *Syst. Biol.* **64**, 505–524 (2015).
3. Merrill, R. M. *et al.* The diversification of Heliconius butterflies: What have we learned in 150 years? *J. Evol. Biol.* **28**, 1417–1438 (2015).
4. Mallet, J., Beltran, M., Neukirchen, W. & Linares, M. Natural hybridization in heliconiine butterflies: the species boundary as a continuum. *Bmc Evol. Biol.* **7**, (2007).
5. Kozak, K. M., Joron, M., McMillan, W. O. & Jiggins, C. D. Rampant genome-wide admixture across the Heliconius radiation. *Genome Biol. Evol.* (2021).
6. Rosser, N., Phillimore, A. B., Huertas, B., Willmott, K. R. & Mallet, J. Testing historical explanations for gradients in species richness in heliconiine butterflies of tropical America. *Biol. J. Linn. Soc.* **105**, 479–497 (2012).
7. Rueda-M, N., Salgado-Roa, F. C., Gantiva-Q, C., Pardo-Diaz, C. & Salazar, C. Environmental drivers of diversification and hybridization in Neotropical butterflies. *Front. Ecol. Evol.* 746 (2021).
8. Loecher, M. & Loecher, M. M. Package ‘RgoogleMaps’. (2020).
9. de Castro, É. C. P., Zagrobelny, M., Cardoso, M. Z. & Bak, S. The arms race between heliconiine butterflies and Passiflora plants – new insights on an ancient subject. *Biol. Rev.* (2018) doi:10.1111/brv.12357.
10. Pinheiro, C. E. G. & Martins, M. Palatability of seven butterfly species (Nymphalidae) to two tyrant-flycatchers in Brazil. *J. Lepidopterists Soc.* **46**, 77–79 (1992).
11. Montgomery, S. H. & Merrill, R. M. Divergence in brain composition during the early stages of ecological specialisation in Heliconius butterflies. *J. Evol. Biol.* n/a-n/a (2016) doi:10.1111/jeb.13027.
12. Ehrlich, P. R. & Gilbert, L. E. Population structure and dynamics of the tropical butterfly Heliconius ethilla. *Biotropica* 69–82 (1973).
13. Young, F. J. & Montgomery, S. H. Pollen feeding in Heliconius butterflies: the singular evolution of an adaptive suite. *Proc. R. Soc. B Biol. Sci.* **287**, 20201304 (2020).
14. Mallet, J. Dispersal and gene flow in a butterfly with home range behavior: Heliconius erato (Lepidoptera: Nymphalidae). *Oecologia* (1986) doi:10.1007/BF00384789.
15. Nadeau, N. J. Genes controlling mimetic colour pattern variation in butterflies. *Curr. Opin. Insect Sci.* **17**, 24–31 (2016).
16. Meier, J. I. *et al.* Haplotype tagging reveals parallel formation of hybrid races in two butterfly species. *Proc. Natl. Acad. Sci.* **118**, (2021).
17. Montejo-Kovacevich, G. *et al.* Genomics of altitude-associated wing shape in two tropical butterflies. *Mol. Ecol.* **0**, (2021).
18. Jiggins, C. D., Salazar, P. A. & Montejo-Kovacevich, G. Heliconiine Butterfly Collection Records from University of Cambridge. Department of Zoology, Cambridge. (2019).
19. Grömping, U. Relative importance for linear regression in *R* : The package **relaimpo**. *J. Stat. Softw.* (2006) doi:10.18637/jss.v017.i01.
20. Hollister, J. & Tarak Shah. *elevatr: Access elevation data from various APIs*. http://github.com/usepa/elevatr (2017).
21. Meier, J. I. *et al.* Haplotype tagging reveals parallel formation of hybrid races in two butterfly species. *Accept. Proc. Natl. Acad. Sci. U. S. Am.* 2020.05.25.113688 (2021) doi:10.1101/2020.05.25.113688.
22. Malinsky, M., Matschiner, M. & Svardal, H. Dsuite-Fast D-statistics and related admixture evidence from VCF files. *Mol. Ecol. Resour.* **21**, 584–595 (2021).
23. Li, H. *et al.* The Sequence Alignment/Map format and SAMtools. *Bioinforma. Oxf. Engl.* **25**, 2078–2079 (2009).
