# Supplementary material for: Repeated genetic adaptation to altitude in two tropical butterflies
Source: Nat Commun. 2022 Aug 9;13:4676. doi: 10.1038/s41467-022-32316-x (PMC9363431; doi:10.1038/s41467-022-32316-x)
Supplement: Supplementary file 2 — Description of Additional Supplementary Files [file 41467_2022_32316_MOESM2_ESM.pdf]

Description of Additional Supplementary Files

File Name: Supplementary Data 1
Description: Table with sample and sequence information of all individuals included in this study.
