# Supplementary material for: Repeated genetic adaptation to altitude in two tropical butterflies
Source: Nat Commun. 2022 Aug 9;13:4676. doi: 10.1038/s41467-022-32316-x (PMC9363431; doi:10.1038/s41467-022-32316-x)
Supplement: Supplementary file 4 — Reporting Summary [file 41467_2022_32316_MOESM4_ESM.pdf]

nature portfolio

Corresponding author(s): Montejo-Kovacevich, Gabriela

Last updated by author(s): 2022/07/20

Reporting Summary

Nature Portfolio wishes to improve the reproducibility of the work that we publish. This form provides structure for consistency and transparency in reporting. For further information on Nature Portfolio policies, see our Editorial Policies and the Editorial Policy Checklist.

Statistics

For all statistical analyses, confirm that the following items are present in the figure legend, table legend, main text, or Methods section.

n/a	Confirmed	
☐	☒	The exact sample size (*n*) for each experimental group/condition, given as a discrete number and unit of measurement
☒	☐	A statement on whether measurements were taken from distinct samples or whether the same sample was measured repeatedly
☐	☒	The statistical test(s) used AND whether they are one- or two-sided<br>*Only common tests should be described solely by name; describe more complex techniques in the Methods section.*
☐	☒	A description of all covariates tested
☐	☒	A description of any assumptions or corrections, such as tests of normality and adjustment for multiple comparisons
☐	☒	A full description of the statistical parameters including central tendency (e.g. means) or other basic estimates (e.g. regression coefficient) AND variation (e.g. standard deviation) or associated estimates of uncertainty (e.g. confidence intervals)
☐	☒	For null hypothesis testing, the test statistic (e.g. *F*, *t*, *r*) with confidence intervals, effect sizes, degrees of freedom and *P* value noted<br>*Give P values as exact values whenever suitable.*
☒	☐	For Bayesian analysis, information on the choice of priors and Markov chain Monte Carlo settings
☐	☒	For hierarchical and complex designs, identification of the appropriate level for tests and full reporting of outcomes
☐	☒	Estimates of effect sizes (e.g. Cohen's *d*, Pearson's *r*), indicating how they were calculated

Our web collection on statistics for biologists contains articles on many of the points above.

Software and code

Policy information about availability of computer code

Data collection	Data collection was not performed with any software.
Data analysis	All non-genomic analyses were run in R V2.13 (R Development Core Team 2011)<br>bwa mem (v. 0.7.15 Li, 2013)<br>samtools (v. 1.9 Li et al., 2009)<br>Picard tools (v 1.92 Broad Institute, 2018)<br>ANGSD (v. 0.933 Korneliussen et al., 2014)<br>GATK (v. 3.7 McKenna et al., 2010)<br>vcftools (v. 0.1.15 Danecek · 2011)<br>R: topodistance (v. 1 Wang 2020), BEDASSLE (v. 1.0), ggplot2 (v. 3.3.6), ntervals (v. 0.15.2), relaimpo (v. 2.2)<br>liftover utility (UCSC Hinrichs, 2006).<br>PCAngsd (v. 0.98)<br>RgoogleMaps (v. 1.4.5.3)<br>Dsuite (v. 0.5 Malinsky et al., 2021)<br>plink (v. 1.07 Chang et al., 2015)<br>RAxML (v. 8.2.9)<br>ABBABABAwindows.py script in https://github.com/simonhmartin/genomics_general.<br><br>Scripts and pipelines have been made available in the public repository Zenodo (DOI https://doi.org/10.5281/zenodo.6836103).

For manuscripts utilizing custom algorithms or software that are central to the research but not yet described in published literature, software must be made available to editors and reviewers. We strongly encourage code deposition in a community repository (e.g. GitHub). See the Nature Portfolio guidelines for submitting code & software for further information.

Data

Policy information about availability of data

All manuscripts must include a data availability statement. This statement should provide the following information, where applicable:

- Accession codes, unique identifiers, or web links for publicly available datasets
- A description of any restrictions on data availability
- For clinical datasets or third party data, please ensure that the statement adheres to our policy

The sequence data generated in this study have been deposited in the European Nucleotide Archive database under accession code PRJEB35570 [https://www.ebi.ac.uk/ena/browser/view/PRJEB35570?show=reads] or elsewhere on ENA if obtained from previous studies as specified in Supplementary Data 1 for each individual. Reference genomes for H. erato and H. melpomene were taken from Lepbase v. 4 (H. erato demophoon and H. melpomene version 2.5, respectively). All records associated to the individuals used for this study are available in the Heliconius Earthcape database (https://heliconius.ecdb.io/).

Field-specific reporting

Please select the one below that is the best fit for your research. If you are not sure, read the appropriate sections before making your selection.

☐ Life sciences ☐ Behavioural & social sciences ☒ Ecological, evolutionary & environmental sciences

For a reference copy of the document with all sections, see nature.com/documents/nr-reporting-summary-flat.pdf

Ecological, evolutionary & environmental sciences study design

All studies must disclose on these points even when the disclosure is negative.

Study description	The study comprises whole-genome sequencing of hundreds of butterflies of two species, Heliconius erato and Heliconius melpomene, across 4 transects, 2 in each side of the Andes and replicated in Ecuador and Colombia. Each transect is made up of three populations with at least 8 individuals in each population.
Research sample	Research samples is made up of 518 whole-genomes of Heliconius erato and H. melpomene, newly sequenced for this study, and 85 whole genomes of five close relatives and 31 individuals from outgroups to be able to perform analyses involving trees. Heliconius erato and H. melpoemne samples were collected from a range of altitudes and locations to enable studying adaptation to altitude on both sides of the Andes in three-population replicate transects of Ecuador and Colombia, where possible. Those transects with two populations represent areas that could not be sampled, either because the butterflies no longer exist in those regions because of land-use change or because we could not sample them due to inaccessible/dangerous regions. Sexes are specified in Supplementary Data 1, and we did not bias our sampling by age/sex nor were organisms manipulated.
Sampling strategy	Butterflies were collected with hand nets and their bodies stored in ethanol and their wings inside envelopes within 12h of collection in the wild. In all populations where we had some individuals (i.e. sampled populations), we sequenced 5 individuals at high depth and the rest at medium-low depth. We sequenced as many individuals as we could with the funds/samples available and aimed for equal sequencing across treatment levels.
Data collection	Butterflies were sampled by many collectors, including most of the authors in the study. Precise locality and altitude were recorded with a GPS (usually with a Garmin GAPS) and written down with pen and paper. G.M.K. and I.A.W. reviewed the data and uploaded it to the Earthcape database (https://heliconius.ecdb.io/)
Timing and spatial scale	Sampling of butterflies was usually carried out between 9am and 4pm and precise locations recorded. Collections took place between 2000-2018. Specific dates for each individual are recorded in the Earthcape database (https://heliconius.ecdb.io/) and on Supplementary Data 1. Gaps in collection period represent different researchers and grants.
Data exclusions	Some individuals were excluded if they were found to be outliers in PCAs, for instance two individuals showed evidence of being hybrids with other subspecies - which is sometimes impossible to determine with the phenotypes alone. 13 individuals were excluded as they had been misidentified in the field and were in fact not Heliconius erato nor Heliconius melpomene.
Reproducibility	This study does not contain experiments.
Randomization	There are no groupings of samples other than natural populations.
Blinding	Random permutations were performed in many of the analyses, in which investigators have no power over group allocation or data subsampling. During sample collection blinding is not possible as investigators know in which location they are and when selecting invidivuals for sequencing investigators need to know the origin of the samples, but they do not know what their genomes will look like (i.e. no possible bias towards any genotype).

Did the study involve field work? ☒ Yes ☐ No

Field work, collection and transport

Field conditions	Fieldwork was carried out across many regions in Ecuador and Colombia. Rainfall and temperatures varies significantly across elevations and an in-depth analyses of microclimates in most of the Ecuadorian populations in this study can be found in a previous study by the authors: Montejo-Kovacevich et al. 2020 "Microclimate buffering and thermal tolerance across elevations in a tropical butterfly". A summary of Ecuadorian climates obtained from WorldClim2 can be found below:<br>Location, precipitation (mm), mean annual temperature (Celsius)<br>highland west (1208m), 2845, 20.2<br>lowland west (468), 2623, 23.1<br>highland east (1219), 4040, 19.3<br>lowland east (420), 3746, 23.6
Location	Exact locations of populations can be found in the Supplementary Information Table S1 and localities of individuals can be found in Supplementary Data 1.
Access & import/export	In Colombia, field collections were conducted under permit no. 530 issued by the Autoridad Nacional de 539 Licencias Ambientales of Colombia (ANLA). In Ecuador, collections during November-December 2011, and September-October 2012, were done under permit 0033-FAU-MAE-DPO-PNY and exported under permits 001-FAU-MAE-DPO-PNY and 006-EXP-CIEN-FAU-DPO-PNY. Permits were obtained from Parque Nacional Yasuní, Ministerio Del Ambiente, La Dirección Provincial de Orellana. Collections in Ecuador during 2017-2019 were conducted under the permit provided by the Ministerio del Ambiente, Ecuador (MAE-DNB-CM-2017-0058).
Disturbance	No disturbance to ecosystems was caused by this study. These butterflies are very abundant and have ~7 generations per year. We only collected individuals from species under study.

Reporting for specific materials, systems and methods

We require information from authors about some types of materials, experimental systems and methods used in many studies. Here, indicate whether each material, system or method listed is relevant to your study. If you are not sure if a list item applies to your research, read the appropriate section before selecting a response.

Materials & experimental systems

n/a	Involved in the study
☒	☐ Antibodies
☒	☐ Eukaryotic cell lines
☒	☐ Palaeontology and archaeology
☐	☒ Animals and other organisms
☒	☐ Human research participants
☒	☐ Clinical data
☒	☐ Dual use research of concern

Methods

n/a	Involved in the study
☒	☐ ChIP-seq
☒	☐ Flow cytometry
☒	☐ MRI-based neuroimaging

Animals and other organisms

Policy information about studies involving animals; ARRIVE guidelines recommended for reporting animal research

Laboratory animals	No animals were kept in laboratories.
Wild animals	Heliconius butterflies were collected with hand nets and precise location recorded, and stored in envelopes until they could be processed in the evening. Butterflies were killed on the same day as they were collected by compressing the thorax, as it is the fastest method available in field sites. All detached wings were photographed with a DSLR camera with a 100 mm macro lens in standardised conditions, images and full records with data are stored in the EarthCape database (https://Heliconius.ecdb.io, Jiggins et al., 2019). Butterfly bodies were stored in pure ethanol, and transferred to a -80C at the earliest possibility, often two or three weeks post-collection. Age of butterflies cannot be determined.
Field-collected samples	No butterflies collected in the field were kept in the laboratory for this study.
Ethics oversight	No ethical approval is required for working with arthropods.

Note that full information on the approval of the study protocol must also be provided in the manuscript.
